# The genome sequence of the gastric gland parasite, *Cryptosporidium proliferans*

**DOI:** 10.1016/j.isci.2026.115597

**Published:** 2026-04-06

**Authors:** Monika M. Wiśniewska, Lenka Tůmová, Jeffrey D. Silberman, Eric D. Salomaki, Joseph Bielawski, Petr Táborský, Lenka Hlásková, Bohumil Sak, Martin Kváč, Martin Kolísko

**Affiliations:** 1 Institute of Parasitology, Biology Centre of the Czech Academy of Sciences, České Budějovice, Czech Republic; 2 Department of Molecular Biology and Genetics, Faculty of Science, University of South Bohemia, České Budějovice, Czech Republic; 3 Department of Animal Husbandry, Faculty of Agriculture and Technology, University of South Bohemia, České Budějovice, Czech Republic; 4 Center for Computational Biology of Human Disease and Center for Computation and Visualization, Brown University, Providence, RI, USA; 5 Department of Biology, Department of Mathematics and Statistics, Dalhousie University, Halifax, Nova Scotia, Canada; 6 Department of Zoology, Faculty of Science, Charles University, Prague, Czech Republic

**Keywords:** Health sciences, Biological sciences, Parasitology, Microbial genomics, Microbiology parasite

## Abstract

*Cryptosporidium* spp. infect humans and many other animals, with a clade of species that targets intestinal enterocytes and a clade that colonizes the gastric gland. Unlike intestinal species, gastric species are understudied and genomic sequences are currently available from *C. muris* and *C. andersoni*. These species cause mild, self-limiting infections, unlike *C. proliferans*, which induces severe and sometimes fatal disease. To investigate genomic differences underlying this pathology, we sequenced the *C. proliferans* genome using short- and long-read technology, producing the most complete gastric *Cryptosporidium* assembly. The genome is intron rich and encodes host invasion genes (e.g., thioredoxin, insulinase/M16-peptidase) near telomeric or subtelomeric regions, which are enriched with G-quadruplex motifs that likely drive protein diversification. Comparative analyses revealed strong synteny among gastric species but only partial synteny with intestinal species. Selective pressure analyses (*d*N/*dS*) showed marked differences between *C. muris* and *C. proliferans*, potentially explaining their contrasting disease outcomes.

## Highlights

• Sequenced a near-complete genome of a highly virulent gastric parasite
• Gastric species exhibit strong synteny but diverge from intestinal species
• G4s motifs generally co-localize with telomeric repeats
• Gene selection may facilitate different virulence phenotypes

## Graphical abstract

**Figure:**
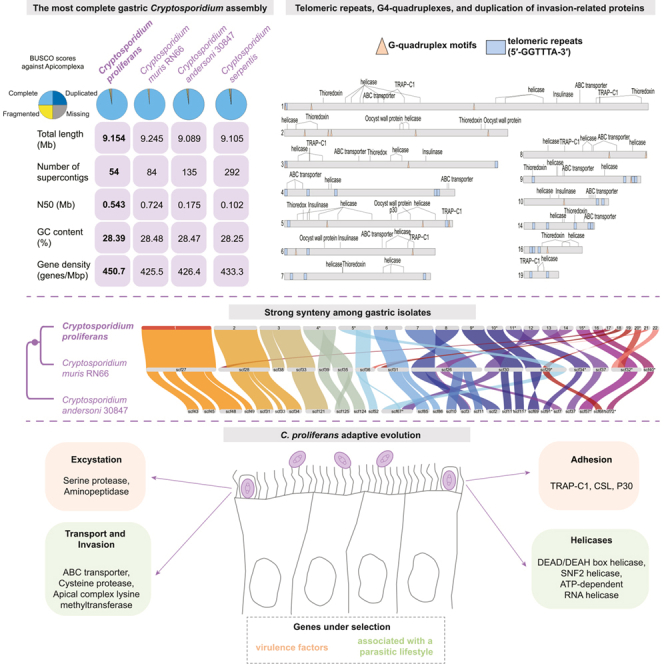

## Introduction

*Cryptosporidium* is a genus of widespread parasitic single-cell eukaryotic organisms that infect a variety of animals across the globe. These apicomplexan parasites specifically target the digestive tract of their hosts, leading to a condition known as cryptosporidiosis. The severity of the disease can range from mild or asymptomatic to life-threatening, largely depending on the species involved and the host’s overall health and immune status. In most healthy people, the infection resolves on its own, but it is considered a serious condition for immunocompromised patients.^1^^,^^2^

*Cryptosporidium* is divided into two primary groups based on the specific region of the host’s digestive tract they reside. One group targets enterocytes that line the intestine, whereas the other invades the gastric gland.^1^ These two groups form distinct clades in phylogenetic analyses.^3^^,^^4^^,^^5^ Compared to the intestinal group, far fewer gastric *Cryptosporidium* species have been described (only 12 valid species described to date^6^^,^^7^^,^^8^^,^^9^^,^^10^^,^^11^^,^^12^^,^^13^^,^^14^^,^^15^^,^^16^). Among these, 4 species have been identified in fish (*C. molnari*, *C. huwi*, *C. abrahamseni*, and *C. bollandi*),^17^ one in amphibians (*C. fragile*),^10^ two in reptiles (*C. varanii* and *C. serpentis*),^3^^,^^18^ two in birds (*C. galli* and *C. proventriculi*),^3^^,^^9^ and three in mammals—namely *C. muris*,^16^ *C. andersoni*,^13^ and *C. proliferans*^6^).

*Cryptosporidium proliferans* was originally isolated from the gastric gland of a naturally infected East African rodent (*Tachyoryctes splendens*) in Kenya. It was initially (mis)identified as *Cryptosporidiummuris* isolate TS03 but was later recognized as a distinct species based on genetic and biological differences from other members of the genus.^6^ Notably, *C. proliferans* is an extremely virulent infectious agent of the gastric gland that causes life-long infections in mice and *Mastomys* spp. and can lead to severe weight loss and massive enlargement of the gastric mucosa.^6^ This sets it apart from other known gastric species that infect mammals, in which infection in most hosts appears to resolve asymptomatically.

*Cryptosporidium* species manipulate the host cell to envelop them within its apical cell membrane, shielding the parasite from the host’s immune response and reducing the likelihood of premature excretion in feces.^19^^,^^20^^,^^21^ These parasites deploy a variety of mechanisms to ensure successful infection by activation of virulence factors that enhance their ability to invade and survive within the host.^22^^,^^23^^,^^24^^,^^25^^,^^26^^,^^27^^,^^28^ Virulence factors are the key in determining the parasite’s pathogenicity, including its ability to infect, damage, and replicate within the host. Several *Cryptosporidium* virulence factors have been identified, each playing a role in specific life cycle stages, such as oocyst excystation (e.g., aminopeptidase and serine protease), sporozoite motility (e.g., thrombospondin-related adhesive protein C1 [TRAP-C1]), attachment and invasion of epithelial cell (e.g., Gp40, Gp60, Gp900, Cpa135, and CpSUB), parasitophorous vacuole formation (e.g., CpABC), host-cell damage (e.g., phospholipases and proteases), and detoxification and nutrient uptake (ATP-binding cassette [ABC] transporters).^23^^,^^29^^,^^30^^,^^31^^,^^32^^,^^33^^,^^34^^,^^35^ Additional enzymes, including hemolysin H4, insulinase, and insulinase-like M16 proteases and helicases with different domains (DEAD-box, DEAH-box, and SNF2) have also been suggested to play a role in virulence.^36^^,^^37^^,^^38^^,^^39^^,^^40^ The vast majority of studies on virulence factors have been conducted on the intestinal species *C*. *parvum*^41^^,^^42^^,^^43^^,^^44^^,^^45^^,^^46^ and far less information is available about the identity and role of virulency factors in gastric-infecting species.

Due to their significant impact on public health and veterinary medicine, intestinal *Cryptosporidium* species have been targeted in research.^47^^,^^48^ To date, genomic data publicly available for gastric *Cryptosporidium* species are restricted to *C. muris* and *C. andersoni*^49^ and the recently sequenced *C. serpentis*.^18^ Genomic data from the highly pathogenic *C. proliferans* are conspicuously missing. In this study, we present the nearly complete genome of *C. proliferans*, obtained through a combination of short- and long-read sequencing technologies. Comparative genomic analyses were conducted looking at *C. proliferans* and the ten *Cryptosporidium* genomes currently available in the CryptoDB.org database (http://CryptoDB.org) and other select apicomplexans (see Table S1). To gain deeper insights into mechanisms that may be involved in the high virulence of *C. proliferans*, we specifically focused on differences among individual gastric *Cryptosporidium* species and between gastric and intestinal species. Additionally, in the genomes of gastric isolates, we computationally identified G-quadruplex motifs (G4s) and regions of contigs containing a high density of telomeric repeats. G4s are non-canonical secondary DNA or RNA structures that form in guanine-rich sequences and are broadly conserved across eukaryotes and prokaryotes. In other systems, G4s are often enriched in telomeres, promoters, and untranslated regions (UTRs), where they can influence processes such as transcription, replication, translation, and genome stability.^50^^,^^51^

Collectively, these analyses establish a comprehensive genomic framework for *C. proliferans*, addressing a significant gap in the currently available data for gastric *Cryptosporidium* species. By integrating comparative genomics with structural motif profiling, this work provides new insights into sequence features that may underlie the exceptional virulence of *C. proliferans*. The resulting dataset offers a valuable resource for future investigations aimed at elucidating molecular mechanisms of host-parasite interactions, genome organization, and potential targets for therapeutic intervention.

## Results

### Genome of *C. proliferans*

**Figure 1:**
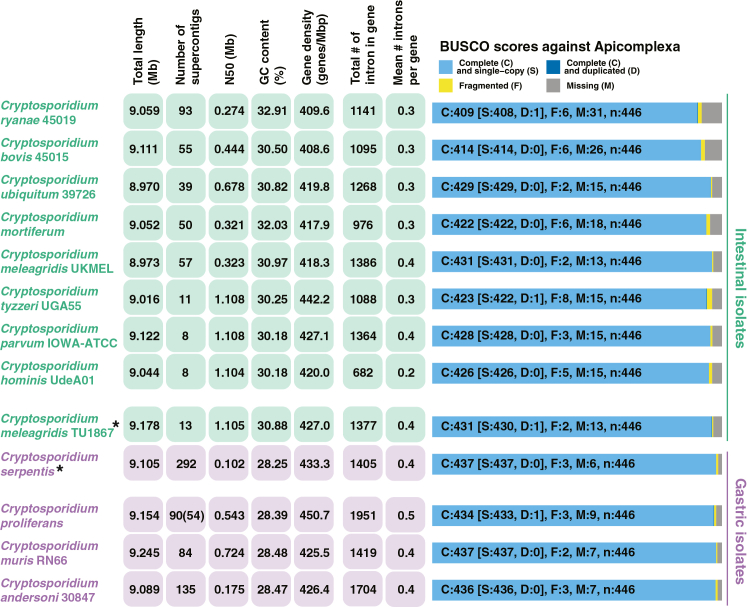
Comparative analysis of *Cryptosporidium* genomes used in this study Summary statistics and BUSCO scores across *Cryptosporidium* datasets. Asterisks denote datasets that were made publicly available after submission of the paper and were therefore not included in the downstream analyses.

**Figure 2:**
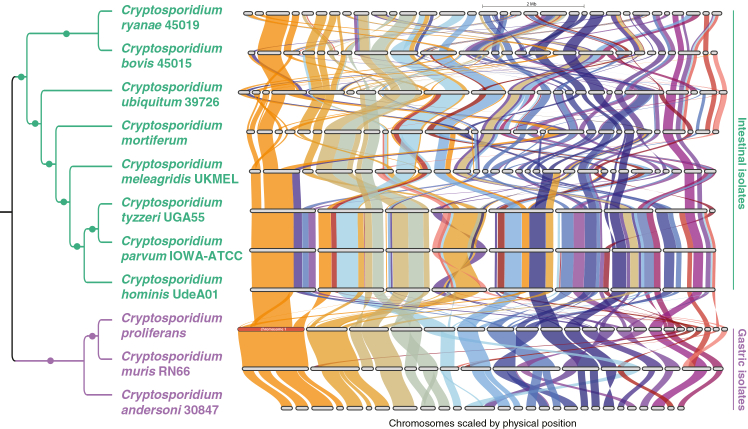
Phylogenetic relationships and gene synteny among *Cryptosporidium* species Cladogram of *Cryptosporidium* species and the synteny among the *Cryptosporidium* spp. genes using *Cryptosporidium proliferans* as the reference genome. The *C. proliferans* full chromosome number 1 is denoted by the red bar. Dots denote 100% bootstrap support.

The assembled nuclear *C. proliferans* genome is haploid, as supported by the minimal number of polymorphic sites (detected by ploidyNGS, see Figure S1), and has a total size of 9.15 Mb with a GC content of 28.39% (Figure 1). It has 167× coverage by short reads and 3× coverage by long reads. The assembly is nearly complete and 99.92% of expressed RNA transcripts mapped along their entire length at ≥95% identity. Benchmarking Universal Single-Copy Orthologs (BUSCO) v.4.1.4^52^ analysis using the apicomplexan reference dataset (apicomplexa_odb10.2019-11-21) indicates high completeness, identifying 97.3% of expected genes (433 single copy and one duplicated; Figure 1). The initial *C. proliferans* genome assembly consisted of 90 contigs. Of these, 54 range in size from 1.3 Mb to 181 bp and contain more than 99.5% of the total genome sequence and coding content. The remaining 36 contigs were excluded from downstream analyses because they contained only 21 predicted genes which either had identical copies encoded on one of the 54 contigs kept in the assembly or contained no coding genes and mostly repetitive sequences (Figure S2). The full assembly, including all contigs and predicted genes, is provided through GenBank: JBPZWI010000000 and Figshare repository: https://doi.org/10.6084/m9.figshare.26057647.^53^ Gene predictions made by a combination of the PASA pipeline v.2.5.2^54^ and AUGUSTUS v.3.3.2^55^ revealed 4,105 protein-coding genes (without pseudogenes) and a total of 1,951 introns (1,135 genes with and 2,970 genes without introns), with a gene density of 450.7 genes/Mbp and 0.5 introns per gene (Table S1). Slightly more than 78% of the genome is exonic, with the average intergenic region measuring 473.16 bp, and an average intron size of 104 bp (Figures 1 and S3). Mapping of RNA sequencing (RNA-seq) data to the genome confirms expression of all predicted proteins. At 98.5% identity of the predicted coding DNA sequence (CDS), *C. proliferans* shares the highest genetic identity with *C. muris* (Figure S4). At least one contig in the assembly (denoted by red bar and white number 1 in the Figure 2) corresponds to an entire chromosome including telomeric regions.

### Synteny among *Cryptosporidium* genomes

*Cryptosporidium muris* and *C. andersoni* genomes showed substantial synteny with each other and the *C. proliferans* genome (Figure 2). A detailed comparison between *C. proliferans* and *C. muris* indicated that the 22 largest contigs from *C. proliferans* (covering 95% of its genome) were highly syntenic with 15 contigs from the *C. muris* genome, accounting for 98.5% of its genome. The most conserved region was observed between contig 1 of *C. proliferans* and “scl27” of *C. muris*, with only minor rearrangements involving genes predominantly annotated as hypothetical proteins.

A high level of synteny was also identified among genomes of *C. tyzzeri* UGA55, *C. parvum* IOWA, and *C. hominis* UdeA01, with only minor gene rearrangements observed on chromosomes labeled “ctyz4”, “19”, and “ln877950” in Figure 2. Other intestinal isolates exhibit lower synteny; in part, this is likely due to incomplete genome assemblies. Although there are some regions with similarities between gastric and intestinal species, there are numerous rearrangements between syntenic blocks (Figure 2, more detailed synteny analysis is included in the Figshare repository^53^).

### Phylogenomics

We performed maximum likelihood (ML) phylogenomic analysis on protein sequences comprising 178 genes from 24 apicomplexan taxa (Figures S5 and S6) rooted with two Chrompodellid taxa—*Chromera velia* and *Vitrella brassicaformis*, and the closely related taxon *Piridium sociabile*. The apicomplexans formed two groups: a “Core Apicomplexan” clade with 100% bootstrap (BS) support and *Cryptosporidium*-Porosporidae clade with 76% BS support. The genus *Cryptosporidium* formed a fully supported clade (100% BS), as did the division of intestinal and gastric species (Figure S5).

### Gene content of *Cryptosporidium* spp.

**Figure 3:**
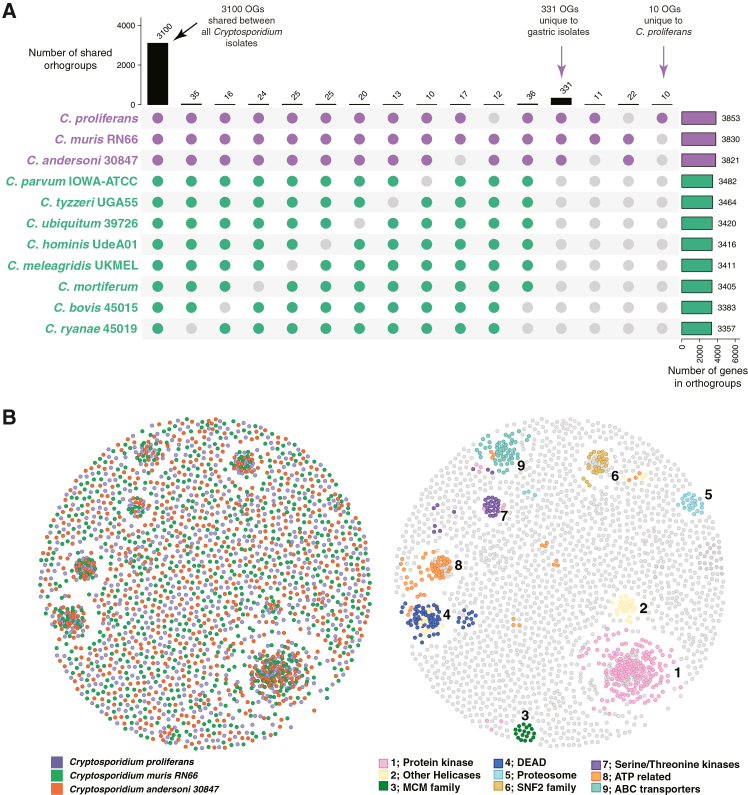
Orthogroup sharing and protein similarity networks among gastric *Cryptosporidium* species (A) Upset plot showing OGs shared among all the *Cryptosporidium* species (3,100), OGs unique to the gastric isolates (331), and unique to *C. proliferans* (10). Gastric annotations are in purple and intestinal in green. Filled colored dots are orthogroups presence and gray dots are orthogroups that are absent. (B) The relationship among proteins in *C. proliferans*, *C. muris* RN66, and *C. andersoni* 30847 was visualized using Gephi v.0.10 with Fruchterman-Reingold layout based on the BLASTP homology results, with pident ≥30% over an overlap of at least 100 amino acids.

Orthofinder v.2.5.4^56^ was used to infer orthogroups (OGs) among the 11 *Cryptosporidium* species used in phylogenomic analysis. This analysis predicted 331 orthogroups unique to the gastric isolates (Figure 3A). Functional enrichment analysis revealed that mitochondrial metabolic processes, such as the tricarboxylic acid (TCA) cycle and oxidative phosphorylation, as well as virulence-related functions, including insulinase activity are overrepresented in these 331 OGs (Figure S6). Ten OGs comprising 28 unique proteins were initially identified as exclusive to *C. proliferans*. However, subsequent tBLASTn searches against the genomic sequences of *C. andersoni* and *C. muris* revealed homologous gene sequences in both species. These results indicate that the genes are in fact present in all three genomes.

Protein network analysis was performed using the Fruchterman-Reingold algorithm as implemented in Gephi v.0.10^57^ on proteins of the three gastric *Cryptosporidium* isolates. Nine large clusters were identified and functionally annotated (Figure 3B). The largest cluster (cluster 1) is enriched in protein kinases. Successively smaller clusters include 4 distinct clusters that were associated with helicase families, including those containing HA2 (cluster 2), DEAD (cluster 4), and SNF2 (cluster 6) domains, as well as minichromosome maintenance complex DNA helicases (cluster 3). Cluster 7 contained serine/threonine kinases, while cluster 8 included genes with ATP-binding domains and ATPase activity. ABC transporters were grouped in cluster 9. Cluster 5 contained proteasome subunit alpha.

**Figure 4:**
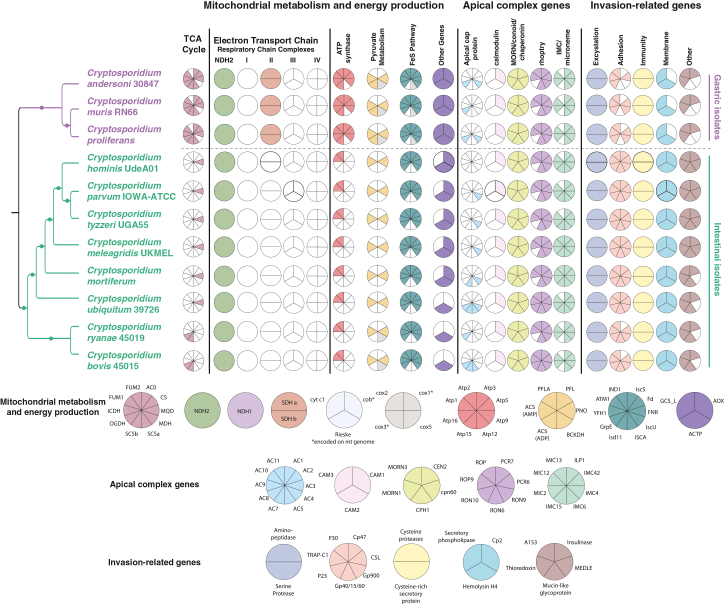
Presence and absence of mitochondrial, apical complex, and invasion-related proteins across *Cryptosporidium* species Cladogram of *Cryptosporidium* species with mapped presence/absence of proteins involved in mitochondrial metabolism (TCA cycle, electron transport chain, pyruvate metabolism, Fe-S cluster pathway, and additional metabolic genes), apical complex genes, and invasion-related genes. Light gray sectors in the Coulson plots indicate the presence of at least one subunit of the branched-chain α-keto acid dehydrogenase complex (BCKDH) or the iron-sulfur cluster assembly (ISCA) pathway. Dots denote 100% bootstrap support.

*In-silico* analyses show that mitochondrial metabolism of *C. proliferans* appears to be identical to that of *C. muris* and *C. andersoni*. The metabolic capacity of the mitochondrial-related organelles of all three gastric species is highly reduced but retains more metabolic functionality than intestinal *Cryptosporidium* species. Each gastric species possesses a partial TCA cycle, NDH2, complex II of the respiratory chain and an almost complete ATP-synthase (complex V). In contrast, most intestinal *Cryptosporidium* species lack all components of the electron transport chain, TCA cycle, and ATP-synthase, except NDH2, ATP1, ATP2, and MQO. The exceptions are the two earliest branching intestinal species, *C. ryanae* and *C. bovis*, that each retained two extra enzymes of the TCA cycle—oxoglutarate dehydrogenase (OGDH) and SCSb—while missing MQO (Figure 4). All sequenced *Cryptosporidium* species have enzymes for anaerobic pyruvate metabolism and substrate level phosphorylation. Alternative oxidase (AOX) was identified in all *Cryptosporidium* species except *C. ubiquitum*, *C. ryanae*, and *C. bovis*. The gastric species, as well as the two intestinal species (*C. ryanae* and *C. bovis*), have dihydrolipoamide dehydrogenase, and only gastric species possess ADP-forming acetyl-CoA synthetase (ACS-ADP).

We searched for 19 known *Cryptosporidium* virulence factors (Figures 4 and S7–S25) and 32 proteins known to be part of the apical complex (Figure 4, Table S2). Membrane occupation and recognition nexus repeat proteins (MORN1 and MORN3), Centrin 2 (CEN2), chaperonin CPN60, and conoid protein hub 1 (CPH1), which are essential for the structural integrity of the conoid, were present in all *Cryptosporidium* datasets as well as calmodulin-like protein 1 (CAM1).

All analyzed *Cryptosporidium* genomes contained at least one apical cap protein, except for *C. hominis*. Rhoptry proteins (ROP, ROP9, and ROP10), rhoptry neck proteins (RON6 and RON9), and preconoidal ring proteins (PCR6 and PCR7) were identified in all *Cryptosporidium* datasets. Rhoptry neck protein 10 (RON10) was found exclusively in two intestinal isolates, *C. ubiquitum* and *C. bovis*. Six microneme-associated proteins were identified in the gastric isolates, and the intestinal *C. ryanae* and *C. bovis* have one or two additional microneme proteins not found in the other intestinal isolates (Figure 4, Table S2).

The presence/absence pattern of 19 invasion related genes shows that gastric species are missing *p23*, *gp40/15/60*, *gp900*, and *cp47* genes encoding adhesion proteins, as well as gene for Mucin-like glycoprotein and Cp2. The two deepest branching intestinal species (*C. bovis* and *C. ryanae*) are also missing *cp47*, and *C. ryanae* is also missing *gp40/15/60*. MEDLE is present in four intestinal species, *C. hominis*, *C. parvum*, *C. tyzzeri*, and *C. meleagridis*^58^ but not in the gastric species. Other adhesion- and invasion-related proteins, serine proteases, cysteine proteases, and cysteine-rich secretory proteins, CSL zinc-finger domain-containing protein, TRAP-C1, P30*,* A153, insulinase, and thioredoxin were conserved among all species analyzed (Figure 4, Table S2). It is important to note that all the virulence factors and genes involved in mitochondrial metabolism were identified in *Cryptosporidium* genomes based on *in-silico* predictions and BLASTP-based homology searches described in detail in the STAR Methods section.

### *C. proliferans* adaptative evolution

**Figure 5:**
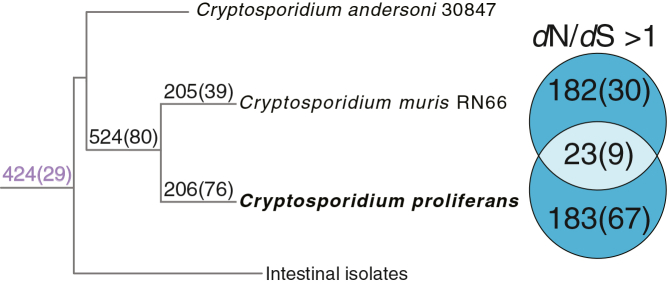
Genes under selective pressure in gastric *Cryptosporidium* species Cladogram of gastric *Cryptosporidium* isolates. Purple colored numbers above the branch leading to gastric isolates indicate genes under unique selective pressure in the gastric isolates (424 total), 29 of which were associated with parasitic lifestyle functions. Numbers above all branches denote the total number of genes under selective pressure with a *d*N/*d*S ratio >1. The Venn diagram adjacent to the branches leading to *C. muris* and *C. proliferans* illustrates the shared and species-specific sets of genes under positive selection (*d*N/*d*S > 1). Numbers in parentheses indicate genes with *d*N/*d*S > 1 that were also significantly supported by a LRT (Chi-square threshold = 6.635, corresponding to 1 degree of freedom and α = 0.01).

The non-synonymous substitution rate (*d*N)/synonymous substitution rate (*d*S) ratio serves as a measure of selective pressure: values equal to 1 are consistent with neutral evolution, values greater than 1 suggest positive selection, and values less than 1 indicate purifying (negative) selection.^59^ We tested for large-scale divergence between gastric and intestinal species in the intensity of natural selection acting on their protein-coding genes. Specifically, we used a likelihood ratio test (LRT) to compare a model having a single *d*N/*d*S ratio for all phylogenetic branches to a model having independent *d*N/*d*S ratios for gastric and intestinal clades. This LRT revealed 424 genes under unique selective pressure in the gastric isolates (after Bonferroni correction for multiple tests with α = 0.001) (Figure 5, Table S3). Among these were 29 genes associated with a parasitic lifestyle, including members of the ABC transporter family, various helicases (DEAD-box, DEAH-box, and SNF2-like), oocyst wall proteins, and TRAP-C1 (genes indicated by the lavender color in Table S3).

For all orthogroups, we used codon models with branch-specific *d*N/*d*S parameters for the *C. muris* and the *C. proliferans* branches, and where possible their common clade, to obtain ML estimates of species-specific selection pressure. Although we used the LRT to verify statistical significance of these *d*N/*d*S estimates, our purpose, here, was to generate and explore novel hypotheses about lineage- and function-specific selection intensity. We did not seek to test an *a-priori* hypothesis on a gene-by-gene basis because formulating the research question in that way would negatively impact the power of the analyses. We found that *C. proliferans* genes had significantly higher *d*N/*d*S estimates than those of *C. muris* (Wilcoxon rank-sum test of aggregated data: *p* value <0.001). Among the analyzed 3,314 OGs, 23 OGs exhibited *d*N/*d*S ratios greater than one in both species, suggesting a shared history of positive Darwinian selection for these genes. Of these, 9 OGs were significantly supported by the LRT for divergent selection pressure (α = 0.01 with one degree of freedom). In species-specific comparisons, we found many genes had *d*N/*d*S estimates >1 but far fewer were significant (threshold α = 0.01) for lineage-specific selection pressure (Figure 5). This is in part due the large estimation errors associated with lineages-specific estimates of *d*N/*d*S relative to the effect size between lineages. In *C. proliferans*, just 67 of the 183 OGs with *d*N/*d*S > 1 were significant for lineage-specific selection pressure. In *C. muris*, 30 of the 182 OGs were significant for divergent selection pressure. The functional annotations for these genes were explored. In *C. proliferans*, one of OGs with *d*N/*d*S > 1 is a known virulence factor, and 10 others are likely associated with a parasitic lifestyle based on the literature (Table S4). In *C. muris*, 7 OGs with *d*N/*d*S > 1 are annotated as virulence factors and another 7 are likely associated with parasitic traits. Along the branch leading to the clade comprised of *C. proliferans* and *C. muris*, 524 OGs have *d*N/*d*S > 1, of which 80 were significant for divergence from the background selection pressure. Among these, 12 are recognized virulence factors and 39 are likely associated with a parasitic lifestyle (Table S4A).

### Telomeric repeats, G4s, and duplication of the invasion-related proteins

**Figure 6:**
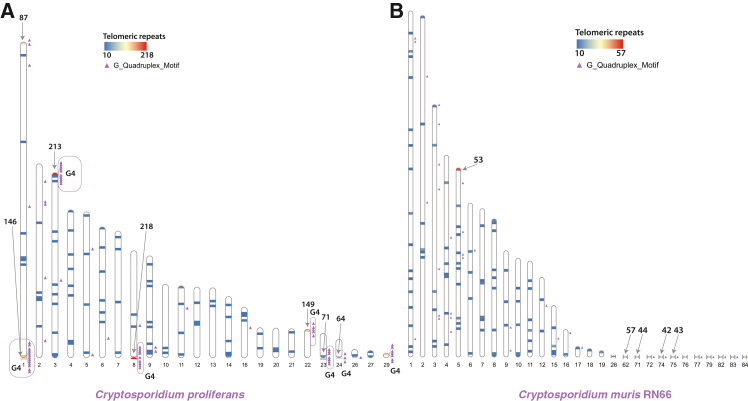
Telomeric repeats and G-quadruplexes in gastric *Cryptosporidium* genomes The placement of the telomeric repeats (colored lines) and the G-quadruplexes (marked as a purple triangle on the side) alongside the longest contigs of (A) *C. proliferans* and (B) *C. muris* genomic assemblies. Arrow at the end of contigs indicates the number of telomeric repeats present.

We identified telomeric repeats (5′-GGTTTA-3′)^60^ in the genomes of gastric species (Figure 6). The *C. proliferans* genome possessed a higher number of telomeric repeats than the genomes of *C. muris* and *C. andersoni* (Figure 6, Table S5. Telomeric repeat analysis of the C. proliferans genome, Table S6. Telomeric repeat analysis of the C. muris genome, Table S7. Telomeric repeat analysis of the C. andersoni genome). We also predicted several G-quadruplex structures in all three gastric species and determined their placement in their genome (Figure 6, Table S8. Identification of G-quadruplex structures in the C. proliferans genome, Table S9. Identification of G-quadruplex structures in the C. muris genome, Table S10. Identification of G-quadruplex structures in the C. andersoni genome). We found a higher abundance of predicted G4s in *C. proliferans* than in *C. muris* and *C. andersoni* (77 G4s in *C. proliferans* (Table S8C), 23 G4s in *C. muris* (Table S9C), and 25 G4s in *C. andersoni* (Table S10C)). Approximately 90% of all predicted G4 in *C. proliferans* were located within 1 kb of regions enriched in telomeric repeats (see Table S8C for the position of G4 clusters supported by both methods), indicating a strong spatial coupling between G4 formation and telomere-associated sequences. Only a small fraction (±10%) of predicted G4 were situated within internal chromosomal regions, suggesting that while G4 structures can occur within chromosome bodies, they are markedly concentrated near telomeric regions in *C. proliferans.*

**Figure 7:**
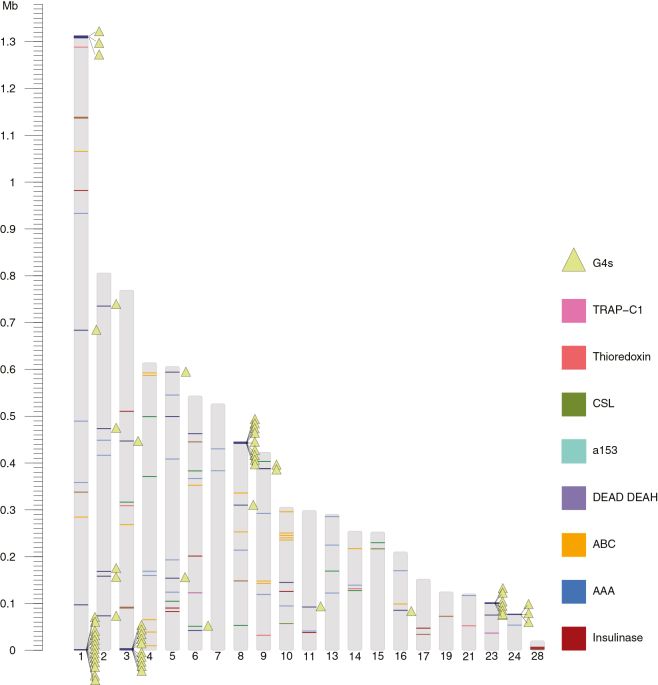
Distribution of virulence factors and G-quadruplexes in the *C. proliferans* genome Genomic distribution of selected virulence factor genes and predicted G-quadruplex structures across contigs of the *C. proliferans* genome assembly.

Analysis of subtelomeric regions in *C. proliferans* revealed multiple duplicated gene families, including insulinase-like proteases, TRAP-C1, and ABC family transporters (Figure 7). These duplicated loci were typically positioned within 0.5 Mb of telomeric repeat clusters and within 0.1 Mb of predicted G4s. In addition to these expanded gene families, approximately 3.8% of genes encoded within subtelomeric regions were annotated as hypothetical proteins, reflecting the presence of poorly characterized loci within these dynamic genomic compartments. Figure 7 shows the organization and copy numbers of these gene families, highlighting their repeated subtelomeric placement.

## Discussion

### *C. proliferans* genome

To date, 11 *Cryptosporidium* genomes have been published, eight from intestinal species and three from gastric species. These assemblies vary widely in completeness, ranging from highly fragmented drafts to complete chromosome-level genomes (Figure 1). Studies combining long- and short-read DNA sequencing technologies have demonstrated substantial improvements in assembly quality.^61^^,^^62^ Here, we sequenced the nuclear genome of stomach infecting *C. proliferans*. Using a combination of Illumina short reads and Oxford Nanopore long reads, we assembled a high-quality and nearly complete *C. proliferans* genome. Among the two published gastric *Cryptosporidium* genomes, *C. andersoni*, is highly fragmented (135 contigs), whereas *C. muris* has been assembled into 84 contigs. In contrast, the final *C. proliferans* assembly contains only 54 (90) contigs, making it one of the most continuous assembly of a published gastric *Cryptosporidium* species to date. This highlights the effectiveness of Oxford Nanopore sequencing, even at relatively low (±3×) coverage when combined with Illumina short-read data.

We identified at least one contig with telomeric repeats at both ends, which is indicative of a complete chromosome (see contig 1 Figure 6). We also detected telomeric signals at one end of 14 additional contigs. *C*. *muris* and *C. proliferans* share 98.5% nucleotide identity over the coding portion of their genomes (Figure S4), consistent with recent divergence into two separate species. Gene prediction analysis identified 4,105 protein-coding genes in *C. proliferans*, including 1,951 introns, making it the most intron-rich *Cryptosporidium* characterized to date.

The genomes of gastric species exhibit a high degree of synteny, especially between the high-quality genomes of *C. muris* and *C. proliferans*. This pattern is comparable to the synteny observed among the chromosome-level assemblies of the closely related intestinal species *C. tyzzeri* UGA55, *C. parvum* IOWA, and *C. hominis* UdeA01.^63^^,^^64^ In contrast, synteny, among this clade and other intestinal species, is more limited and becomes increasingly discordant when comparing gastric and intestinal *Cryptosporidium* species. Overall, comparative analyses reveal high synteny among gastric species and moderate synteny between gastric and intestinal species.

### Gene content evolution and metabolic capacity within *Cryptosporidium* species

Orthofinder^56^ analyses identified roughly 300 orthogroups unique to the gastric species (Figure 3). As shown previously, many of these OGs are associated with mitochondrial metabolism. This is consistent with the observation that gastric *Cryptosporidium* species*,* including *C. proliferans*, possess a more complex mitochondrial metabolism than intestinal species^4^^,^^37^ (Figure 4). Some orthogroups exclusive to gastric species also include known virulency factors, such as insulinases from the M16 peptidase family, which differs from those found in intestinal species.

Some patterns of gene presence and absence in the early-diverging intestinal species *C. ryanae* and *C. bovis* resemble those of gastric species more than those of other intestinal taxa. For example, they lack MIC13, IMC15, and cp47, yet retain OGDH and SCSb. Given that *C. ryanae* and *C. bovis* form the deepest branch within intestinal species, these patterns may represent the ancestral state of the intestinal clade.^65^ It is also important to note that our results and observed patterns are based on the current state of the individual genome assemblies and predicted protein sequences.

Orthofinder^56^ analysis revealed no variation in orthogroup presence among gastric species as the 10 orthogroups initially identified as unique to *C. proliferans* are also present in other gastric species genomes and were simply mis-predicted. Moreover, greater than 99% of the orthogroups show patterns consistent with vertical inheritance.

### Subtelomeric regions and G4s

Subtelomeric regions are among the most dynamic and unstable parts of chromosomes. They range in size from ∼0.1 Mb in yeast to ∼0.5 Mb in humans and undergo substantial rearrangements, including segmental duplications.^66^^,^^67^ These regions promote phenotypic polymorphism, affecting both coding and non-coding RNA expression, and contribute significantly to genome plasticity.^67^^,^^68^^,^^69^

In *Cryptosporidium* spp., subtelomeric regions likely facilitate gene expansion through duplication events, providing a reservoir for diversification and the emergence of novel gene functions.^37^^,^^65^^,^^70^ Prior work in *C. parvum*, for example, has shown that duplication of MEDLE and insulinase genes may enhance host adaptability and broaden host range.^70^ We hypothesize that, similar to *C. parvum*, the subtelomeric regions in *C. proliferans* might also act as hotspots for gene duplication, potentially driving the emergence of lineage-specific traits and contributing to ecological and host-range diversification. However, this is based purely on the observations that subtelomeric regions in *C. proliferans* also contain duplicated virulence-associated genes, for example insulinase-like proteases, TRAP-C1, and ABC family transporters (Figure 7).

We detected telomeric repeats at the termini of 14 contigs in the *C. proliferans* genome, suggesting that these represent complete chromosomal ends. The number of telomeric repeats is higher in *C. proliferans* than in *C. muris* or *C. andersoni*. For example, *C. proliferans* contig 1 extends much further at both ends than its homolog in *C. muris* (contig 27), with an additional ∼1,400 bp of repeats at one end and ∼5,500 bp at the other end. Illumina-only assemblies used for *C. muris* and *C. andersoni* likely failed to resolve full telomeric regions.

G4s are non-canonical four-stranded DNA or RNA structures formed by guanine-rich sequences and have been documented in a number of eukaryotic genomes. G4 motifs are most often associated with telomeric repeats, where they can influence telomere architecture and telomerase activity^71^^,^^72^^,^^73^^,^^74^^,^^75^ and are proposed to function as protection of telomeres in eukaryotes^76^^,^^77^; such as ciliates (*Stylonychia lemnae*),^78^ yeast (*Saccharomyces cerevisiae*),^79^^,^^80^ and vertebrates, including humans.^77^ G4s can be located in gene promoters, where they may regulate transcription by forming structures that block transcription machinery. They can also occur in UTRs of mRNA, where they influence post-transcriptional processes, such as translation efficiency and mRNA stability. On the other hand, helicases containing DEAD-box and SNF2 domains—both present in *C. proliferans* (Figure 3)—can unwind these structures, relieving G4-dependent transcriptional repression.^81^^,^^82^

G4s have been computationally and experimentally identified in several pathogenic protozoa, including *Plasmodium falciparum*, *Trypanosoma brucei*, and *Leishmania major*.^83^ In *P. falciparum*, their enrichment near hypervariable subtelomeric virulence gene families (notably the *var* genes) led to the hypothesis that G4s contributed to antigenic diversification.^81^^,^^84^ Experimental studies have confirmed nuclear G4 formation, identified G4-binding proteins localized to telomeres and G-rich RNAs, and shown that chemical stabilization or disruption of G4s alters gene expression and parasite viability; highlighting their functional and therapeutic relevance.^85^^,^^86^^,^^87^^,^^88^ However, most of the evidence linking G4s to gene expression regulation and the evolution of virulence factors in pathogens is associative rather than based on experimental validation.^74^^,^^89^ In Apicomplexa, no experimental data have been generated addressing G4-mediated regulation of gene expression or their role in genomic or locus stability.

In the *C. proliferans* genome, we identified a high concentration of G4 structures within 1 kb of telomeric repeat-rich regions. The propensity of G4s to be in close proximity of telomeric repeats in the *C. proliferans* genome suggests that they are involved in telomere protection. In contrast, the *C. muris* genome contains far fewer G4s (Figure 6). However, this is most likely the result of an incomplete assembly of the chromosomal ends due to the technical limitation of short-read data. We have also found multiple instances of predicted G4s that are within 1 kb of duplicated virulence factors (thioredoxin, insulinase-like proteases, AAA and ABC transporters; see Figure 7). Not only is the proximity notable but the greater density of predicted G4 motifs near these genes in *C. proliferans* raises the possibility that local G4 landscapes could influence mutation rates, recombination frequency, or *cis*-regulatory dynamics that accelerate gene diversification in subtelomeric compartments, as has been proposed for *var* genes in *Plasmodium*.^81^^,^^84^ To move beyond correlation, targeted experimental approaches are needed, for example: genome-wide G4 mapping (e.g., G4-seq or BG4 immunoprecipitation), biochemical characterization of potential G4-binding proteins in *Cryptosporidium*, perturbation studies using G4-stabilizing or destabilizing ligands, and assays for subtelomeric recombination or transcriptional changes following manipulation of G4s or relevant helicases.

### Contrasting different parasitic behavior

*Cryptosporidium proliferans* exhibits parasitic behavior that differs considerably from other known gastric *Cryptosporidium* species. In some hosts, *C. proliferans* is associated with chronic, lifelong infections, and pronounced pathological changes,^6^ which are not common traits in other gastric isolates. Comparative genomic analyses of *C. proliferans* and other gastric species show several genetic aspects that might play a role in its unique behavior. One possibility is that the difference in pathogenicity is driven by the presence of several hypothetical genes of unknown function. However, we have not found any genes unique to *C. proliferans* genome. We initially identified ten OGs that appeared to be unique to *C. proliferans*; however, all of these proteins are also present in the genomes of the other two gastric species and were only missed during gene prediction.

Results suggest pervasive divergence in the intensity of natural selection (*d*N/*d*S) acting on gastric and intestinal species (424 genes). Further analysis of *d*N/*d*S ratios between *C. proliferans* and *C. muris* suggest divergence in selection intensity among the gastric species due to lineage-specific effects on a smaller subset of genes. Lineage-specific positive selection in a set of multigene families could indicate that the encoded proteins contribute to functional differences in host specificity and pathogenicity. Similar findings have been shown between some of the intestinal species, such as *C. bovis* and *C. parvum*^65^ and *C. parvum* IOWA and *C. hominis*.^90^ These studies showed that some of the invasion-related protein families, such as insulinase-like peptidases, helicases, and TRAP-C1, as well as ABC transporters have elevated *d*N/*d*S ratio. Moreover, several genes indicated to be under selective pressure in *C. proliferans* and *C. muris* and have known virulency factors as in other *Cryptosporidium* species.^65^^,^^91^^,^^92^ Presence of these genes remains relatively conserved among *Cryptosporidium* genomes. However, sequence polymorphisms within these genes may influence transcription and translation efficiency, potentially contributing to the distinct biological traits observed between gastric and intestinal isolates, as well as between the *Cryptosporidium* spp. in general. Our analyses of *d*N/*d*S ratios across the *C. muris* and *C. proliferans* genomes showed that *C. proliferans* has significantly higher average *d*N/*d*S ratio than *C. muris*, consistent with these proteins in *C. proliferans* evolving at a faster rate than in *C. muris*. Such elevated rates can arise from an overall relaxation of selection pressure (affecting all sites in a gene) in *C. proliferans*, or from a larger fraction of sites evolving by positive selection in *C. proliferans*.^93^ These alternatives can be formally tested in the future by sampling additional lineages of *C. muris* and *C. proliferans* and using appropriately designed codon models.^59^^,^^94^ However, we identified 183 and 182 genes in *C. proliferans* and *C. muris,* respectively, with lineage-specific estimates of *d*N/*d*S > 1 (Figure 5). Many of these genes may be under lineage-specific positive selection and potentially contribute to genome-specific adaptations. Some of these are known virulence factors or are associated with a parasitic lifestyle (Table S4). It is plausible the suite of such genes under positive selective pressure might create enough of a difference in individual protein specificity/functionality to drive differences in the phenotype of parasite behavior. Finally, several known virulence factors and genes involved in parasitic lifestyles are located near G4 structures or within subtelomeric repeat regions areas that are hypothesized to influence gene expression and accelerate evolutionary changes. One aspect we could not explore given our current data is epigenetic regulation, which is also likely to play a role in differentiating the behaviors of *C. proliferans* and *C. muri*s. We believe that a combination of the genetic differences described above likely contributes to the observed phenotypic differences; however the exact specific “modes of action” will likely have to be investigated by detailed studies of expression and function of individual proteins, as well as sampling additional gene sequences and analyzing them with more complex evolutionary models.

### Limitations of the study

A cornerstone of comparative genomic studies such as this is access to high-quality, well-assembled genomes. However, the analyses presented here are inherently influenced by uneven data quality among available *Cryptosporidium* genomes. In particular, gastric species are represented by fewer and generally lower-quality assemblies than intestinal species, many of which were generated using more advanced sequencing strategies. This disparity may have introduced biases in comparisons of gene content, synteny, and structural genomic features, potentially affecting the interpretation of evolutionary patterns. In addition, several conclusions—such as the identification of virulence factors, subtelomeric gene duplications, and G4s—are based primarily on *in-silico* predictions and therefore require experimental validation. Finally, the limited number of available gastric genomes restricts the resolution of evolutionary analyses. Broader taxon sampling together with additional functional data will be necessary to fully resolve the mechanisms underlying the distinct pathogenic behavior of *C. proliferans*.

## Resource availability

### Lead contact

Further information and requests for resources and reagents should be directed to and will be fulfilled by the lead contact, Monika M. Wiśniewska (monika.wisniewska@paru.cas.cz).

### Materials availability

This study did not generate new unique reagents.

### Data and code availability

• The Whole Genome Shotgun project has been deposited at DDBJ/ENA/GenBank: JBPZWI000000000. The version described in this paper is JBPZWI010000000. The genome assembly, Illumina and MinION DNA-seq reads have been deposited at NCBI GeneBank: PRJNA1301312^95^ and are publicly available as of the date of publication. The RNA-seq data generated in this study are deposited in the NCBI Sequence Read Archive: SRR34857600^96^ and are publicly available.
• This paper does not report original code.
• Datasets supporting the conclusions of this article are available in the Figshare repository: https://doi.org/10.6084/m9.figshare.26057647.^53^ Any additional information required to reanalyze the data reported in this paper is available from the lead contact upon request.

## Acknowledgments

We sincerely thank all current members of our laboratory for their continuous support, collegiality, and for fostering such a collaborative, encouraging, and enjoyable working environment. Their commitment, insightful discussions, and willingness to help have greatly enriched both our research and daily lab life. This research was funded by 10.13039/501100001824Czech Science Foundation (GACR 21–23773S) awarded to M. Kváč and Grant Agency of 10.13039/100010100University of South Bohemia (GAJU 005/2022/Z) awarded to L.Tůmová. This study was also supported by the ERD fund “Centre for Research of Pathogenicity and Virulence of Parasites” (no. CZ.02.1.01/0.0/0.0/16_019/0000759) and by IT4Innovations National Super Computer Center (project #Open-34-44) to M. Kolísko.

## Author contributions

M.M.W., M. Kváč, and M. Kolísko conceived the design of the study. M.M.W. conducted the analyses and drafted the manuscript. J.D.S. and L.T. performed nucleic-acid extractions and sequencing. M. Kváč, L.H., and L.T. provided culture samples used in the experiments. E.D.S. and J.B. participated in the analyses. M. Kolísko, M. Kváč, B.S., J.D.S., and P.T. were involved in the writing of the manuscript. All authors have read and approved the final version of the manuscript and agree with the order of presentation of the authors.

## Declaration of interests

The authors declare no competing interest.

## STAR★Methods

### Key resources table

**Table:** 

REAGENT or RESOURCE	SOURCE	IDENTIFIER
**Biological samples**		

oocysts of *Cryptosporidium proliferans*	Institute of Parasitology, Biology Centre of the Academy of Sciences of the Czech Republic, https://www.paru.cas.cz/sekce/veterinarni-lekarska-parazitologie/laborator-veterinarni-a-medicinske-protistologie/	Institute of Parasitology, Biology Centre of the Academy of Sciences of the Czech Republic

**Chemicals, peptides, and recombinant proteins**		

cetyltrimethylammonium bromide (ctab)	Sigma-Aldrich	Cat# 219374-100 GM

**Critical commercial assays**		

RNeasy PowerMicrobiome Kit	QIAGEN	Cat# 26000-50-2
SQK-LSK109 ligation kit	Oxford Nanopore Technologies, Oxford, UK	Cat# SQK-LSK109

**Deposited data**		

The Whole Genome Shotgun project	The Whole Genome Shotgun project has been deposited at DDBJ/ENA/GenBank under the accession JBPZWI000000000. The version described in this paper is version JBPZWI010000000.	GeneBank: https://www.ncbi.nlm.nih.gov/nuccore/JBPZWI000000000.1/
genome assembly, Illumina and MinION DNA-seq reads	NCBI GeneBank under the BioProject number PRJNA1301312	GeneBank: https://www.ncbi.nlm.nih.gov/bioproject/PRJNA1301312/
The RNA-seq reads	NCBI Sequence Read Archive under SRR34857600	NCBI Sequence Read Archive: https://www.ncbi.nlm.nih.gov/bioproject/?term=PRJNA1301286
Datasets supporting the conclusions of this article	Figshare repository	Figshare: https://figshare.com/articles/dataset/Assemblies_and_data_zip/26057647

**Software and algorithms**		

FastQC (version 0.11.9)	Andrews, S. FASTQC. (2010). A quality control tool for high throughput sequence data.^97^	FastQC
Trimmomatic (version 0.39)	Bolger AM, Lohse M, Usadel B. (2014). Trimmomatic: a flexible trimmer for Illumina sequence data. Bioinform. 30(15):2114–20.^98^	Trimmomatic
Guppy (version 4.5.4 + 66c1a77)	Wick RR, Judd LM, Holt KE. (2019). Performance of neural network basecalling tools for Oxford Nanopore sequencing. Genome Biol. 20(1):129.^99^	Guppy
Unicycler (version 0.4.9b)	Wick RR, Judd LM, Gorrie CL, Holt KE. (2017). Unicycler: Resolving bacterial genome assemblies from short and long sequencing reads. PLOS Comput Biol. 13(6):e1005595.^100^	Unicycler
blobtools2	Challis R, Richards E, Rajan J, Cochrane G, Blaxter M. (2020). BlobToolKit – interactive quality assessment of genome assemblies. G3: Genes, Genomes, Genetics. 10(4):1361–74.^101^	blobtools2
minimap2 (version 2.28)	Li H. (2021). New strategies to improve minimap2 alignment accuracy. Bioinform. 37(23):4572–4.^102^	minimap2
ploidyNGS (version 3.1.3)	https://github.com/diriano/ploidyNGS	ploidyNGS
TopHat (version 2.1.1)	Kim D, Pertea G, Trapnell C, Pimentel H, Kelley R, Salzberg SL. (2013). TopHat2: accurate alignment of transcriptomes in the presence of insertions, deletions and gene fusions. Genome Biol. 14(4):R36.^103^	TopHat
Trinity (version 2.10.0)	Haas BJ, Papanicolaou A, Yassour M, Grabherr M, Blood PD, Bowden J et al. (2013). *De novo* transcript sequence reconstruction from RNA-seq using the Trinity platform for reference generation and analysis. Nat Protoc. 8(8):1494–512.^104^	Trinity
TransDecoder (version 5.5.0)	Brian H, Papanicolaou A. Transdecoder (Find Coding Regions Within Transcripts). Available from: http://transdecoder.github.io.^105^	TransDecoder
PASA (version 2.5.2)	Haas BJ, Delcher AL, Mount SM, Wortman JR, Smith RK, Hannick LI et al. (2003). Improving the *Arabidopsis* genome annotation using maximal transcript alignment assemblies. Nucleic Acids Res. 31(19):5654–66.^54^	PASA pipeline
add_intron_features.py	https://github.com/Dalhousie-ICG/icg-shared-scripts	add_intron_features
fix_genes_with_false_introns.py	https://github.com/Dalhousie-ICG/icg-shared-scripts	fix_genes_with_false_introns
validate_gene_models_in_gff3.py	https://github.com/Dalhousie-ICG/icg-shared-scripts	validate_gene_models_in_gff3
Augustus (version 3.3.2)	Stanke M, Keller O, Gunduz I, Hayes A, Waack S, Morgenstern B. (2006). AUGUSTUS: *ab initio* prediction of alternative transcripts. Nucleic Acids Res. 34(suppl_2):W435–9.^55^	Augustus
AGAT (version 0.8.0)	Dainat J. (2022). AGAT: Another Gff Analysis Toolkit to handle annotations in any GTF/GFF format. Available from: https://doi.org/10.5281/zenodo.3552717.^106^	AGAT
ANI calculator	Yoon SH, Ha SM, Lim J, Kwon S, Chun J. (2017). A large-scale evaluation of algorithms to calculate average nucleotide identity. Antonie Van Leeuwenhoek. 110(10):1281–6.^107^	ANI calculator
eggNOG (version 5.0)	Huerta-Cepas J, Szklarczyk D, Heller D, Hernández-Plaza A, Forslund SK, Cook H et al. (2019). eggNOG 5.0: a hierarchical, functionally and phylogenetically annotated orthology resource based on 5090 organisms and 2502 viruses. Nucleic Acids Res. 47(D1):D309–14.^108^	eggNOG
BlastKoala (version 3.0)	Kanehisa M, Sato Y, Morishima K. (2016). BlastKOALA and GhostKOALA: KEGG tools for functional characterization of genome and metagenome sequences. J Mol Biol. 428(4):726–31.^109^	BlastKoala
BUSCO (version 4.1.4)	Manni M, Berkeley MR, Seppey M, Simao FA, Zdobnov EM. (2021). BUSCO Update: Novel and streamlined workflows along with broader and deeper phylogenetic coverage for scoring of eukaryotic, prokaryotic, and viral Genomes. Vol. 38, Mol Biol Evol. 2021.p. 4647–54.^52^	BUSCO
OrthoFinder (version 2.5.4)	Emms DM, Kelly S. (2019). OrthoFinder: phylogenetic orthology inference for comparative genomics. bioRxiv. 466201.^56^	OrthoFinder
topGO (version 4.2.3)	Alexa A, Rahnenfuhrer J. (2010). topGO: enrichment analysis for gene ontology. R package version. 2(0):2010.^110^	topGO
clusterProfiler (version 4.6.2)	Wu T, Hu E, Xu S, Chen M, Guo P, Dai Z et al. (2021). ClusterProfiler 4.0: A universal enrichment tool for interpreting omics data. Innovation (Camb). 2(3):100141.^111^	clusterProfiler
Gephi (version 0.10)	Bastian M, Heymann S, Jacomy M. (2009). Gephi: an open source software for exploring and manipulating networks. Proc Int AAAI Conf Weblogs Soc Media. 3(1):361–2.^57^	Gephi
GENESPACE (version 1.2.3)	Lovell JT, Sreedasyam A, Schranz ME, Wilson M, Carlson JW, Harkess A et al. (2022). GENESPACE tracks regions of interest and gene copy number variation across multiple genomes. Elife. 11:e78526.^112^	GENESPACE
Phylofisher	Tice AK, Žihala D, Pánek T, Jones RE, Salomaki ED, Nenarokov S et al. (2021). PhyloFisher: A phylogenomic package for resolving eukaryotic relationships. PLoS Biol. 19(8):e3001365.^113^	Phylofisher
IQ-TREE (version 1.6.12)	Nguyen LT, Schmidt HA, von Haeseler A, Minh BQ. (2015). IQ-TREE: a fast and effective stochastic algorithm for estimating maximum-likelihood phylogenies. Mol Biol Evol. 32(1):268–74.^114^	IQ-TREE
PREQUAL (version 1.02)	Whelan S, Irisarri I, Burki F. (2018). PREQUAL: detecting non-homologous characters in sets of unaligned homologous sequences. Bioinform. 34(22):3929–30.^115^	PREQUAL
MAFFT (version 7.453)	Katoh K, Misawa K, Kei-ichi K, Miyata T. (2002). MAFFT: a novel method for rapid multiple sequence alignment based on fast Fourier transform. Nucleic Acids Res. 30(14):3059–66.^116^	MAFFT
DIVVIER (version 1.01)	https://github.com/simonwhelan/Divvier	DIVVIER
trimAl (version 1.4.rev22)	Capella-Gutiérrez S, Silla-Martínez JM, Gabaldón T. (2009). trimAl: a tool for automated alignment trimming in large-scale phylogenetic analyses. Bioinform. 25(15):1972–3.^117^	trimAl
RAxML (version 8.2.12)	Stamatakis A. (2014). RAxML version 8: a tool for phylogenetic analysis and post-analysis of large phylogenies. Bioinform. 30(9):1312–3.^118^	RAxML
HMMER (version 3.3)	Johnson LS, Eddy SR, Portugaly E. (2010). Hidden Markov model speed heuristic and iterative HMM search procedure. BMC Bioinform. 11:431.	HMMER
pal2nal (version 14)	Suyama M, Torrents D, Bork P. (2006). PAL2NAL: robust conversion of protein sequence alignments into the corresponding codon alignments. Nucleic Acids Res. 34(Web Server issue):W609-612.^119^	pal2nal
PAML (version 4.8)	Yang Z. (2007). PAML 4: phylogenetic analysis by maximum likelihood. Mol Biol Evol. 24(8):1586–91.^120^	PAML
FMutSel	Yang Z, Nielsen R. (2008). Mutation-Selection Models of Codon Substitution and Their Use to Estimate Selective Strengths on Codon Usage. Mol Biol Evol. 25(3):568–79.^121^	FMutSel
tidk (version 0.2.41)	Brown M, González De la Rosa PM, Mark B. (2023). A telomere identification toolkit. Zenodo; Available from: https://zenodo.org/records/10091385.^122^	tidk
Quadron	https://github.com/abcsFrederick/non-B_gfa	Quadron
Adobe Illustrator (version 28.4.1)	https://www.adobe.com	Adobe
FigTree (version 1.4.4)	https://tree.bio.ed.ac.uk/software/figtree/	FigTree
RStudio (version 2021.09.0)	https://cran.rstudio.com	RStudio
R (version 4.2.2)	https://cran.rstudio.com	R

**Other**		

NCBI	https://www.ncbi.nlm.nih.gov/	NCBI

### Experimental model and study participant details

The *Cryptosporidium proliferans* isolate (formerly known as *C. muris* TS03) was maintained in susceptible laboratory rodents, including severe combined immunodeficient (SCID) mice and southern multimammate mice (*Mastomys coucha*), at the animal facility of the Institute of Parasitology, Biology Center of the Czech Academy of Sciences. Animals were housed under standard laboratory conditions in accordance with Czech legislation (Act No. 246/1992 Coll. on the Protection of Animals Against Cruelty) and relevant European regulations and were supplied with sterilized food and water *ad libitum*. Experimental procedures were approved by the institutional Ethics Committee and the Committee of the Czech Academy of Sciences (protocol no. 73-2024-P).

The parasite was propagated *in vivo* in SCID mice. Fecal samples were stored in 2.5% potassium dichromate at 4°C–8°C until use, and oocysts were recovered from fecal material using the cesium chloride gradient purification method. As *Cryptosporidium* spp. are obligate intracellular parasites, continuous *in vitro* culture is not possible, so propagation was performed in animal hosts.

### Method details

#### Sample collection and sequencing

Approximately 200,000,000 oocysts of *C. proliferans* were purified using sucrose gradient and cesium chloride gradient centrifugation from the fecaes of infected SCID mice and stored in 2.5% potassium dichromate at 4–8°C.^123^ Total genomic DNA was extracted using a modified cetyltrimethylammonium bromide (CTAB) method (modified from Doyle & Doyle, 1987).^124^ Total RNA was isolated following the protocol of the RNeasy PowerMicrobiome Kit (Qiagen, Hilden, Germany).

The gDNA and RNA used for Illumina sequencing was shipped to commercial companies (Macrogen Inc. South Korea for RNA and IAB, Czech Republic for RNA) for TruSeq DNA PCR-free and TruSeq stranded mRNA libraries preparation and sequencing. The libraries were sequenced using the Illumina NovaSeq6000 platform, producing 13 million and 17 million 150 bp paired-end reads for the genomic and transcriptomic data, respectively. Quality check of Illumina data was performed using FastQC v0.11.9^97^ and low-quality regions and adapter sequences were removed using Trimmomatic v0.39^98^ with ‘ILLUMINACLIP:TruSeq3-PE-2.fa:2:30:1 CROP:133 LEADING:30 TRAILING:30’ parameters. We obtained 30,8 million DNA long-reads sequences using the SQK-LSK109 ligation kit for library preparation, which was subsequently loaded onto a MinION flowcell (Oxford Nanopore Technologies, Oxford, UK). Long reads were base-called and trimmed with guppy v4.5.4 + 66c1a77.^97^

#### Genome assembly, gene prediction, and functional annotations

The draft genome of *C. proliferans* was assembled using a hybrid assembly approach with Illumina and MinION reads and Unicycler v0.4.9b.^100^ The assembly was checked for possible contaminations using blobtools2^101^; none was found. All contigs were classified as Apicomplexa or had no hit against the NT Blast database. Illumina DNA-seq reads were mapped to the genome using minimap2 v2.28^102^ to determine the ploidy of the genome with ploidyNGS v3.1.3 (https://github.com/diriano/ploidyNGS). RNA-seq reads were mapped onto the clean genome assembly using TopHat v2.1.1,^103^ generating a bam file for genome-guided transcriptome assembly with Trinity v2.10.0.^104^ Open reading frames were translated with TransDecoder v5.5.0.^105^ The genome-guided transcriptome assembly and genome assembly were fed into the PASA v2.5.2 pipeline.^54^ The resulting model gene structures were validated with in-house python scripts (add_intron_features.py, fix_genes_with_false_introns.py, and validate_gene_models_in_gff3.py from https://github.com/Dalhousie-ICG/icg-shared-scripts) and used to train Augustus v3.3.2.^55^ The hints file gene predictions were generated using the previously generated bam files (after mapping RNA-seq reads to the genome) and proteins (generated by TransDecoder). The Augustus genome annotations were carried out with optimized mode, and introns, RNA-seq and protein hints.

#### Comparative genomics analysis

We used the AGAT v0.8.0^106^ gff analysis toolkit to compute structural characteristics and the ANI calculator^107^ to assess the nucleotide-level identity among the coding regions of the 11 genomes analyzed in this study. To obtain the functional annotations of protein coding genes, CDS sequences were used as a query for eggNOG v5.0 searches with default parameters,^108^ and BlastKoala v3.0 searches against eukaryotic gene models implemented in Kyoto Encyclopedia of Genes and Genomes (KEGG).^109^ Additionally, *C. proliferans* CDS sequences were queries in BLASTP searches against the nr database and a database created using other *Cryptosporidium* spp. genomes. The completeness of the genomes used in this study was assessed using BUSCO v4.1.4^52^ search against eukaryotic (eukaryota_odb10.2019-11-21) and Apicomplexa (apicomplexa_odb10.2019-11-21) datasets. Next, the peptide sequences in all the datasets (Table S1) were used for the OrthoFinder v2.5.4^56^ analyses. The enrichment analyses of the genes in the orthogroups unique to the gastric isolates were performed using terms from Gene Ontology (GO) and the topGO v4.2.3^110^ and Orthologous Groups, KEGG Pathways, and PFAM domains with clusterProfiler v4.6.2.^111^ The relationships among proteins in gastric species (*C. proliferans* strain TS03, *C. muris* strain RN66, and *C. andersoni* isolate 30847) were visualized using Gephi v0.10^57^ with Fruchterman-Reingold layout based on the results of BLASTP homology analysis, with a threshold of pident ≥ 30% over at last 100 amino acids. The syntenic relationships analysis among genomes of 11 *Cryptosporidium* was performed at the gene-level using the orthogroups computed by OrthoFinder and GENESPACE v1.2.3^112^ R library.

#### Phylogenetic and phylogenomic analysis

Phylogenomic analyses were conducted using Phylofisher^113^ with the addition of the predicted protein datasets of three gastric and eight intestinal *Cryptosporidium* species (Table S1) to the existing curated Phylofisher database.^4^^,^^113^ The script fisher.py was used for the identification of potential orthologs; informant.py reported information about the number of potential orthologs recognized for each taxon per gene; next, the working_dataset_constructor.py provided single gene fasta files of all possible orthologs and paralogs for these data and all others in the publicly provided Phylofisher database containing data for multiple taxa from the eukaryotic Tree of Life.

Single gene trees were manually parsed to identify orthologs for inclusion in phylogenomic analyses and were added to the Phylofisher database^113^ following methods described in Mathur et al. 2023 and Terpis et al. 2025.^5^^,^^125^ There were 178 genes left after ortholog identification, from which an ortholog-only dataset was constructed that included *Cryptosporidium* spp. taxa. The matrix_constructor.py Phylofisher script was used to align and concatenate the orthologs under default settings, resulting in the final phylogenomic matrix comprised of 178 genes (77,956 aa sites) and 27 taxa. This dataset was subjected to ML analysis in IQ-TREE v1.6.12 (LG + C60 + F+ G)^114^ with support estimated using 1,000 Posterior Mean Site Frequency (PMSF) bootstrap replicates. Single gene datasets, including all considered sequences (i.e., including orthologs and paralogs) and ortholog-only datasets are available in the Figshare repository.^53^

#### *In silico* predictions of mitochondrial metabolism and virulence factors

To infer mitochondrial metabolic pathways, we selected amino acid sequences of genes of interest based on the previously published studies of Apicomplexa mitochondrial metabolism.^4^ Putative invasion-related factors were identified through a comprehensive literature review,^25^ and representative seed sequences were compiled. These seed sequences were then used as queries in BLASTP searches against *Cryptosporidium* and other Apicomplexa datasets (Table S1), and the NCBI non-redundant (NR) protein database. All hits with an e−value below 1e^−5^ were retrieved and incorporated into initial gene-specific datasets.

Protein datasets for individual genes were cleaned from sequencing errors and non-homologous sites using PREQUAL v1.02,^115^ aligned using MAFFT v7.453^116^ (-globalpair --maxiterate 1000), and filtered for uncertainty and errors using DIVVIER v1.01 (https://github.com/simonwhelan/Divvier) (--partial -mincol 4 -divvygap). Initial trees were computed from the alignments trimmed with trimAl v1.4.rev22 (-gt 0.1)^117^ using IQ-TREE v1.6.12^114^ under the LG + C20 + F + G model, and 1,000 bootstrap replicates for each gene were computed using RAxML v8.2.12 with PROTGAMMALG4X model.^118^ Bootstrap support was mapped to each tree and the resulting trees were then manually inspected to remove paralogous sequences and contamination.

To identify the presence or absence of apical complex protein orthologues, including highly divergent ones, we utilized a slightly modified protocol from Koreny et al. 2021.^126^ First, we created orthogroups from *Cryptosporidium* (including *C. proliferans*) and *Toxoplasma gondii* TM49 genomes using Orthofinder v2.5.4.^56^ We then collected seed sequences from previously identified putative apical complex proteins known from *Cryptosporidium* spp. and *T.* gondii (using the Koreny et al., 2021 datasets)^126^ and selected all orthogroups containing one or more of these sequences for downstream analysis. Each selected orthogroup was aligned using MAFFT-LINSI^116^ and a hmm profile was built using HMMER v3.3.^119^ We then used these hmm profiles to search *Cryptosporidium* genomes and recovered all sequences with an e-value below 1e^−10^. These sequences were then used as queries in reciprocal BLASTP searches against *T. gondii* TM49. Proteins that did not return the intended seed sequence as the top hit were excluded and categorized as “unrelated proteins”.

#### Genes under selective pressure

Genes under selective pressure were recovered from Orthogroups predicted by Orthofinder. In the first step we selected 3,365 OGs (out of 4,632 OGs from Orthofinder results) that contained a single gene copy for all three gastric species and at least three other intestinal species. These were used for the *d*N/*d*S analyses. For aligned proteins within each orthogroup, every amino acid was aligned with the corresponding nucleotide sequences using pal2nal v14^127^ using the codon alignment option. The resulting nucleotide alignments were used to compute trees with RAxML v8.2.12 under GTRGAMMA model^118^ with support values generated from 100 rapid bootstrap replicates. The gene sequences that generated trees with an overall topology inconsistent with the phylogenomic tree were excluded from further analyses.

Selective pressure acting on genes within *Cryptosporidium* spp. genomes was evaluated using codon-based models implemented in CODEML of PAML v4.8.^120^ The trees computed for each orthogroup were used in an LRT comparing the M0 model (a “one-ratio” model assuming a single *d*N/*d*S ratio, across all branches and sites) with a so-called “branch model” (model that allows a subset of branches to evolve under independent *d*N/*d*S ratio). Equilibrium frequencies were modeled according to FMutSel.^121^ LRTs involved testing the likelihood of the data between model M0 and the “branch model” with 1 degree of freedom. Initially we tested and identified genes with a different *d*N/*d*S ratio between gastric and intestinal species, with an alpha-value of 0.001 and Bonferroni correction for multiple comparisons. Secondly, we used CODEML to estimate branch-specific *d*N/*d*S with the “branch model”, where terminal branches of *C. proliferans* and *C. muris* (and if monophyletic the branch leading to the *C. proliferans* and *C. muris* clade) were fit to the data with independent *d*N/*d*S ratios. Here, we also performed LRT to infer statistical significance in the difference between one-ratio vs. specific branch model, but we did not correct for multiple comparisons in order not to lose the ability to mark genes of potential interest for future studies. Wilcoxon sum-rank test was used to carry out post-hoc comparison of the estimated *d*N/*d*S values between *C. proliferans* and *C. muris*.

#### Identification of telomeric repeats and G-quadruplexes

We used the Telomere Identification toolkit (tidk) v0.2.41^122^ to recognize telomeric repeats present in *C. proliferans* genome contigs and to predict subtelomeric regions of the contigs. We used the telomere repeat sequence that is characteristic of *Cryptosporidium* spp. (5′-GGTTTA-3′), as described in Liu et al. 1998^60^ and implemented tidk searches with a window of 10 kb. The tidk search results are depicted on Figure 6 and the full output of the analysis can be found in FigShare repository.^53^ G-quadruplex motifs were annotated on both strands using default parameters with Quadron^128^ and evaluated with the gfa tool (https://github.com/abcsFrederick/non-B_gfa).

### Quantification and statistical analysis

Statistical support for phylogenetic trees was computed by nonparametric bootstrapping. The *d*N/*d*S estimations were performed using likelihood-based codon substitution models implemented in CODEML from the PAML v4.8 package. A LRT was used to calculate statistical significance between the M0 (one-ratio) model with branch models allowing lineage-specific *d*N/*d*S ratios. Significance was assessed with an alpha value set to 0.001 with Bonferroni corrections for comparison of intestinal and gastric species and α = 0.01 without correction for analyses of *d*N/*d*S specific to *C. proliferans* and *C. muris*. Wilcoxon sum-rank test was used for post-hoc comparison of the estimated *d*N/*d*S values between *C. proliferans* and *C. muris*. Gene Ontology and pathway enrichment in topGO v4.2.3 and clusterProfiler v4.6.2 were run with default corrections for multiple testing.

## Supplemental information

Document S1. Figures S1–S25

Table S1. Overview of genomic datasets and assembly quality metrics Summary of datasets used in this study, including BUSCO completeness scores assessed against eukaryotic and apicomplexan lineages. Also includes basic statistics of genome assemblies and intron-related metrics.

Table S2. Distribution of invasion-related and metabolic genes across *Cryptosporidium* genomes Presence/absence and copy number of apical complex proteins, invasion-related genes, and proteins involved in mitochondrial metabolism. Values within table cells indicate copy number, and presence is highlighted in blue.

Table S3. Results of likelihood ratio tests for selection analyses Summary of likelihood ratio test results after Bonferroni correction for multiple testing (α = 0.001). Genes highlighted in lavender are classified as associated with a parasitic lifestyle.

Table S4. Genes under selective pressure in *Cryptosporidium* species Summary of *d*N/*d*S analyses. Rows highlighted in green indicate virulence-related genes and rows in orange denote known virulence factors. (A) Complete set of analyzed genes; (B) genes where *C. proliferans* and *C. muris* form a monophyletic group; (C) genes with *d*N/*d*S > 1 in *C. proliferans* and < 1 in *C. muris*; (D) genes with *d*N/*d*S > 1 in *C. muris* and < 1 in *C. proliferans*; (E) genes with *d*N/*d*S > 1 in both species.

Table S5. Telomeric repeat analysis of the *C. proliferans* genome Summary of detected telomeric repeats and their distribution across contigs in the *C. proliferans* assembly.

Table S6. Telomeric repeat analysis of the *C. muris* genome Summary of detected telomeric repeats and their distribution across contigs in the *C. muris* assembly.

Table S7. Telomeric repeat analysis of the *C. andersoni* genome Summary of detected telomeric repeats and their distribution across contigs in the *C. andersoni* assembly.

Table S8. Identification of G-quadruplex structures in the *C. proliferans* genome Results of G-quadruplex prediction analyses: (A) Quadron results; (B) gfa tool results; (C) overlap between both methods.

Table S9. Identification of G-quadruplex structures in the *C. muris* genome Results of G-quadruplex prediction analyses: (A) Quadron results; (B) gfa tool results; (C) overlap between both methods.

Table S10. Identification of G-quadruplex structures in the *C. andersoni* genome Results of G-quadruplex prediction analyses: (A) Quadron results; (B) gfa tool results; (C) overlap between both methods.
