# Supplementary material for: The genome sequence of the gastric gland parasite, Cryptosporidium proliferans
Source: iScience. 2026 Apr 6;29(5):115597. doi: 10.1016/j.isci.2026.115597 (PMC13125899; doi:10.1016/j.isci.2026.115597)
Supplement: Document S1. Figures S1–S25 [file mmc1.pdf]

iScience, Volume *29*

Supplemental information

The genome sequence of the gastric gland parasite, *Cryptosporidium proliferans*

Monika M. Wiśniewska, Lenka Tůmová, Jeffrey D. Silberman, Eric D. Salomaki, Joseph Bielawski, Petr Táborský, Lenka Hlásková, Bohumil Sak, Martin Kváč, and Martin Kolísko

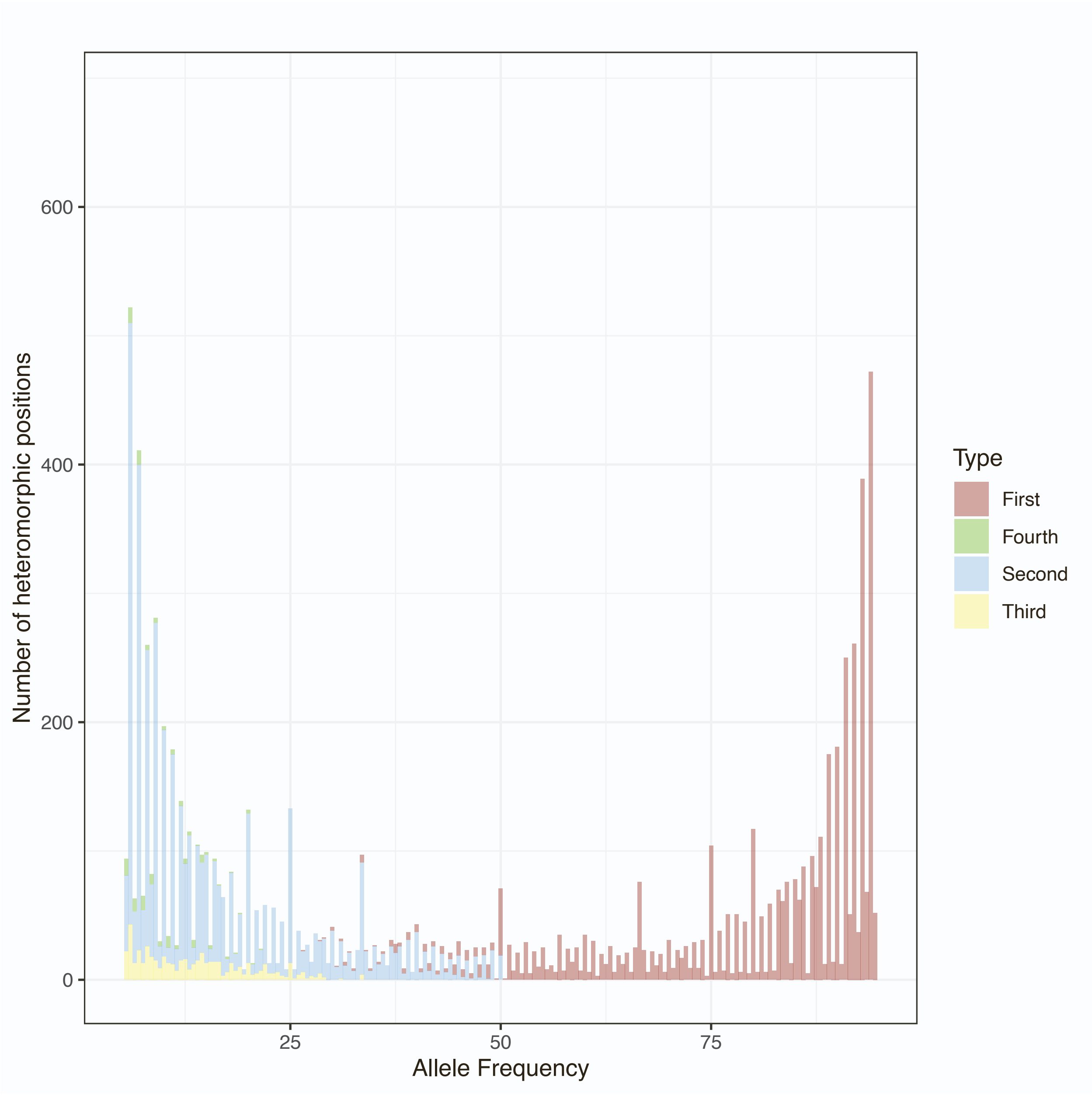

Fig. S1: Ploidy of the Cryptosporidium proliferans genome.
The histogram displays allele frequencies. The x-axis represents the fraction of sequence reads corresponding to each of the four possible alleles, while the y-axis shows the number of heteromorphic (variant) positions. The location of peaks along the x-axis indicates the ploidy level. In the series labeled "First," a peak around 95% corresponds to the most dominant allele, consistent with a haploid genome. Meanwhile, the "Second" series, with a peak near 5%, reflects the second most frequent allele.

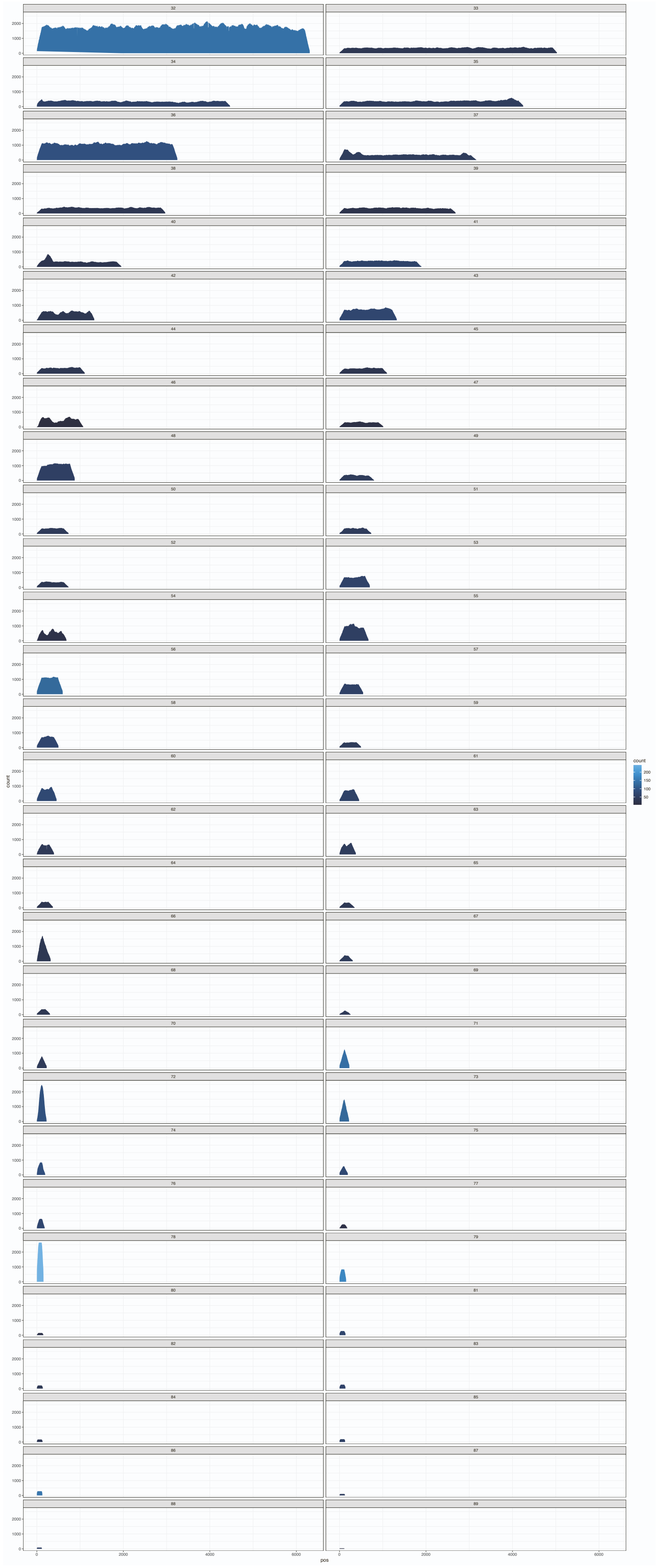

Fig. S2: Mapping of Illumina DNA reads to the 59 contigs of the assembly shorter than 14Kb.
The histograms show the count of the Illumina genomic reads (y-axis) mapped to each position (x-axis) of the 59 contigs. The colors denote the number of reads, the lighter the blue, the more reads have mapped to this position.

Total gene length	7167013 [bp]
Mean gene length	1737 [bp]
Longest gene	41172 [bp]
Shortest gene	119 [bp]

Total intron length	206207 [bp]
Mean intron length	104 [bp]
Longest intron	1094 [bp]
Shortest intron	8 [bp]

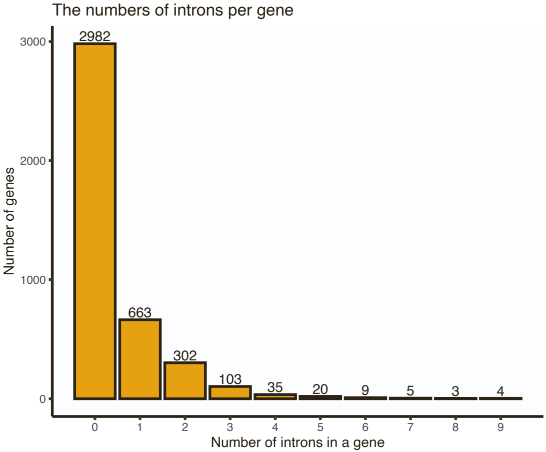

Fig. S3: Additional statistics for the *C. proliferans* genome assembly.

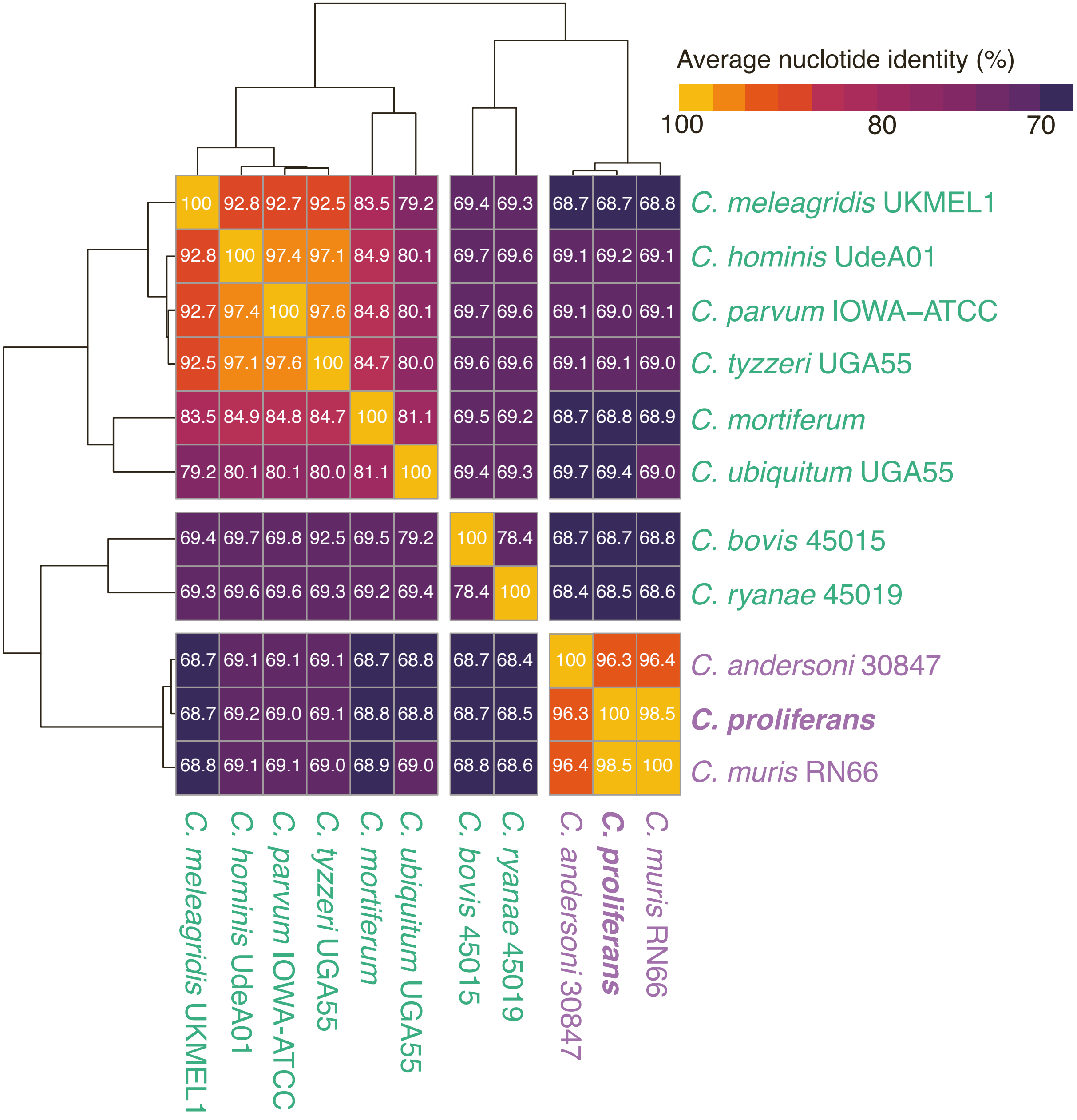

Fig. S4: Average nucleotide identity over coding-sequences of the *Cryptosporidium* genomes used in this study.

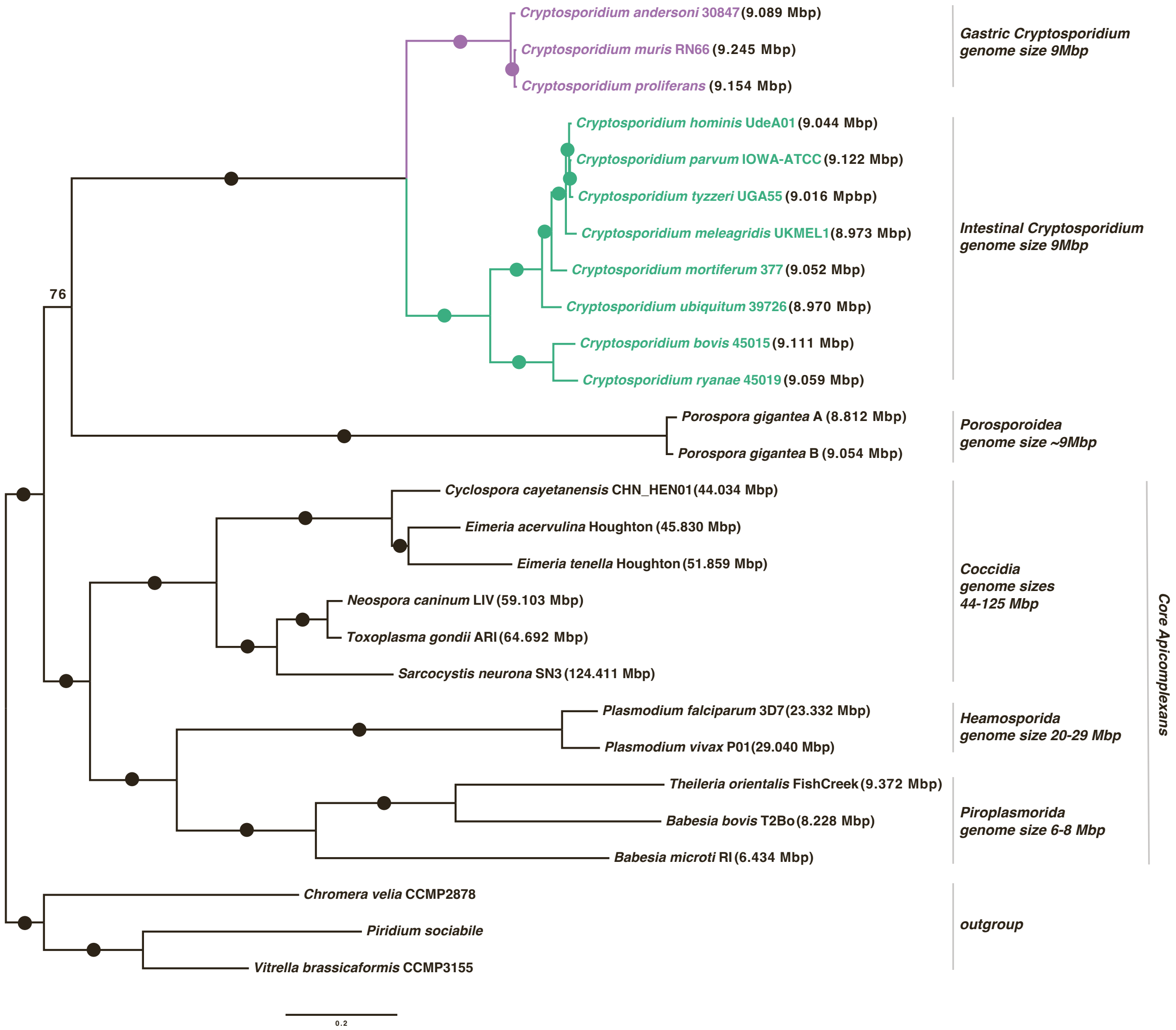

Fig. S5: Maximum Likelihood tree of the apicomplexan datasets used in this study.
The tree was constructed based on 178 genes in IQ-TREE using LG + C60 + F + G model and 1000 PMSF boostraps. The tree was rooted with two Chrompodellid taxa (*Chromera velia*, *Vitrella brassicaformis*) and *Piridium sociabile*. Black dots denote 100% BS support.

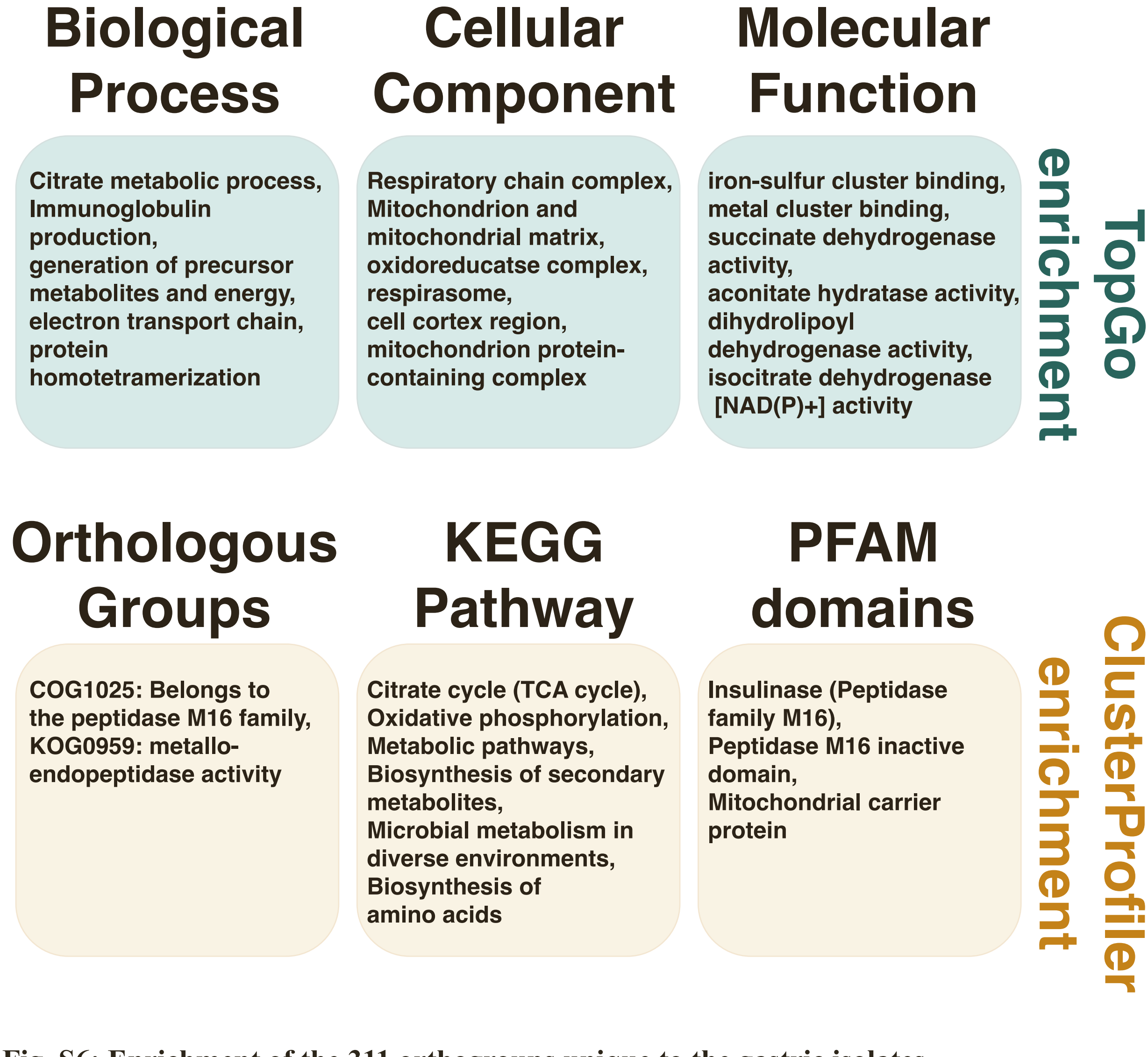

Fig. S6: Enrichment of the 311 orthogroups unique to the gastric isolates.

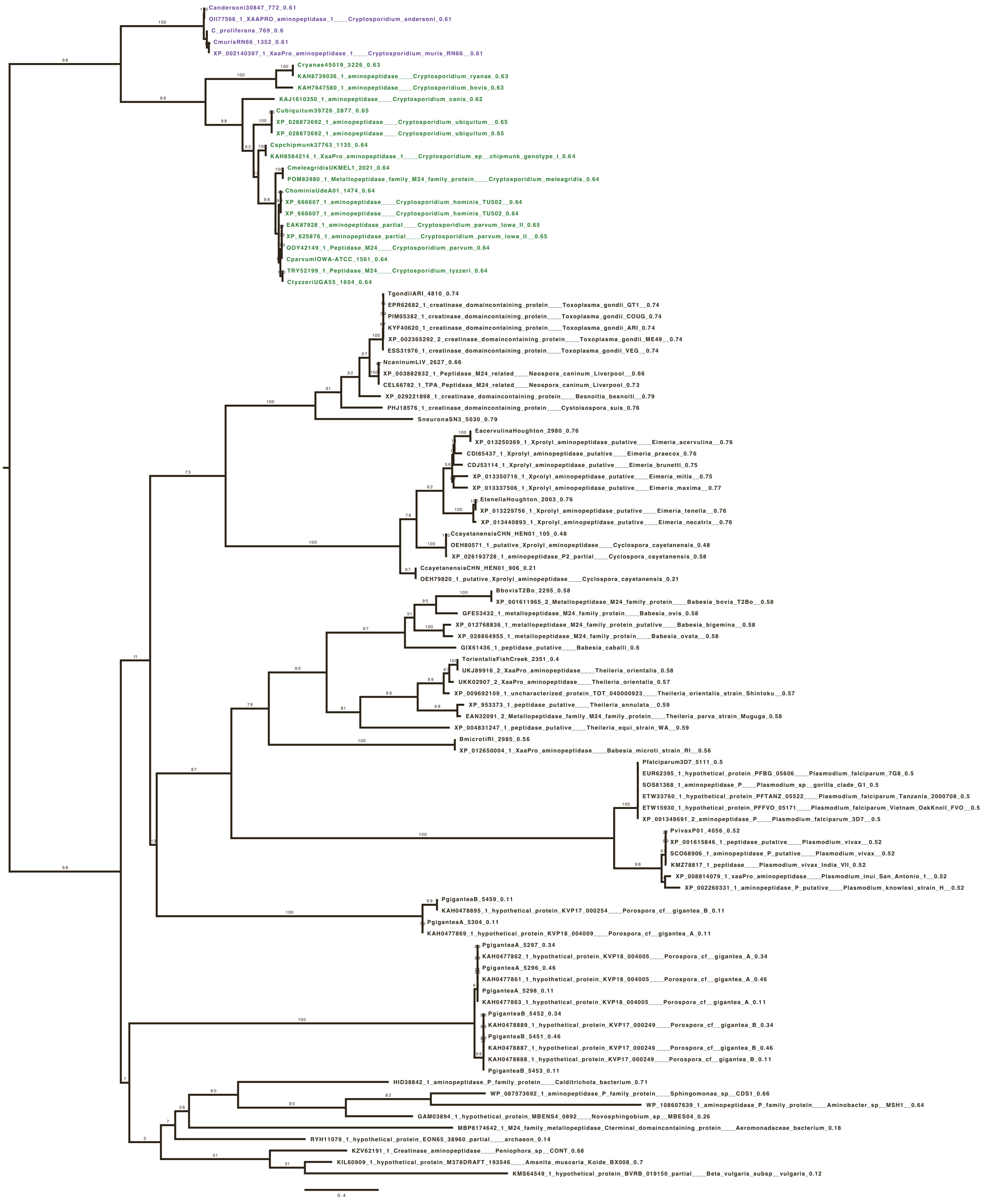

Fig. S7: Maximum likelihood phylogenetic tree of Aminopeptidase sequences.
The tree was inferred using IQ-TREE under the LG+C20+F+G model. Branch support values were estimated from 100 rapid bootstrap replicates calculated in RAxML using the PROTGAMMALG4X model. Gastric *Cryptosporidium* species are highlighted in purple, and intestinal species in green.

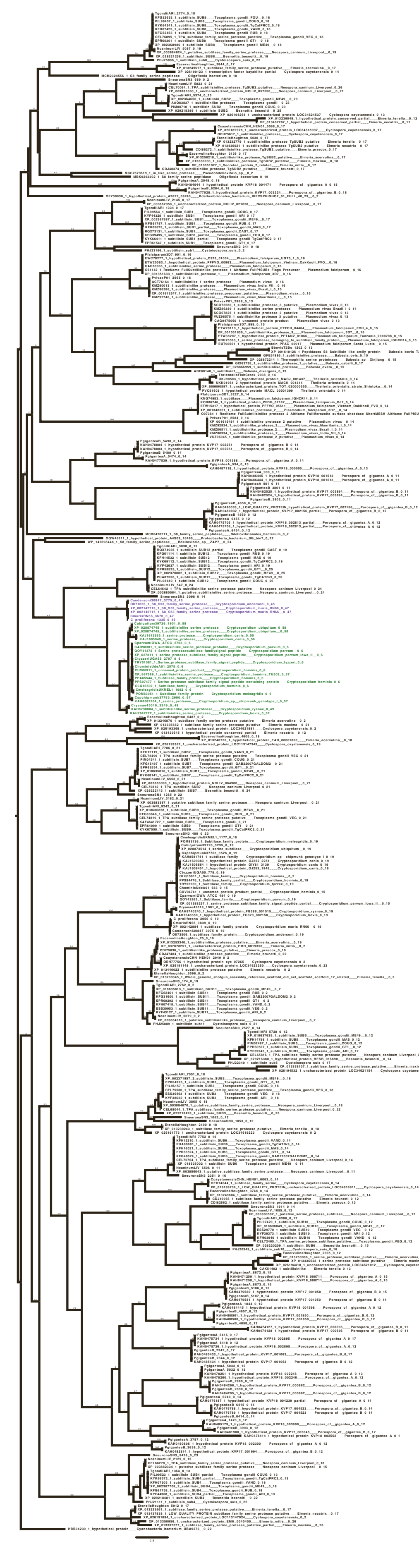

Fig. S8: Maximum likelihood phylogenetic tree of Serine protease sequences.
The tree was inferred using IQ-TREE under the LG+C20+F+G model. Branch support values were estimated from 100 rapid bootstrap replicates calculated in RAxML using the PROTGAMMALG4X model. Gastric *Cryptosporidium* species are highlighted in purple, and intestinal species in green.

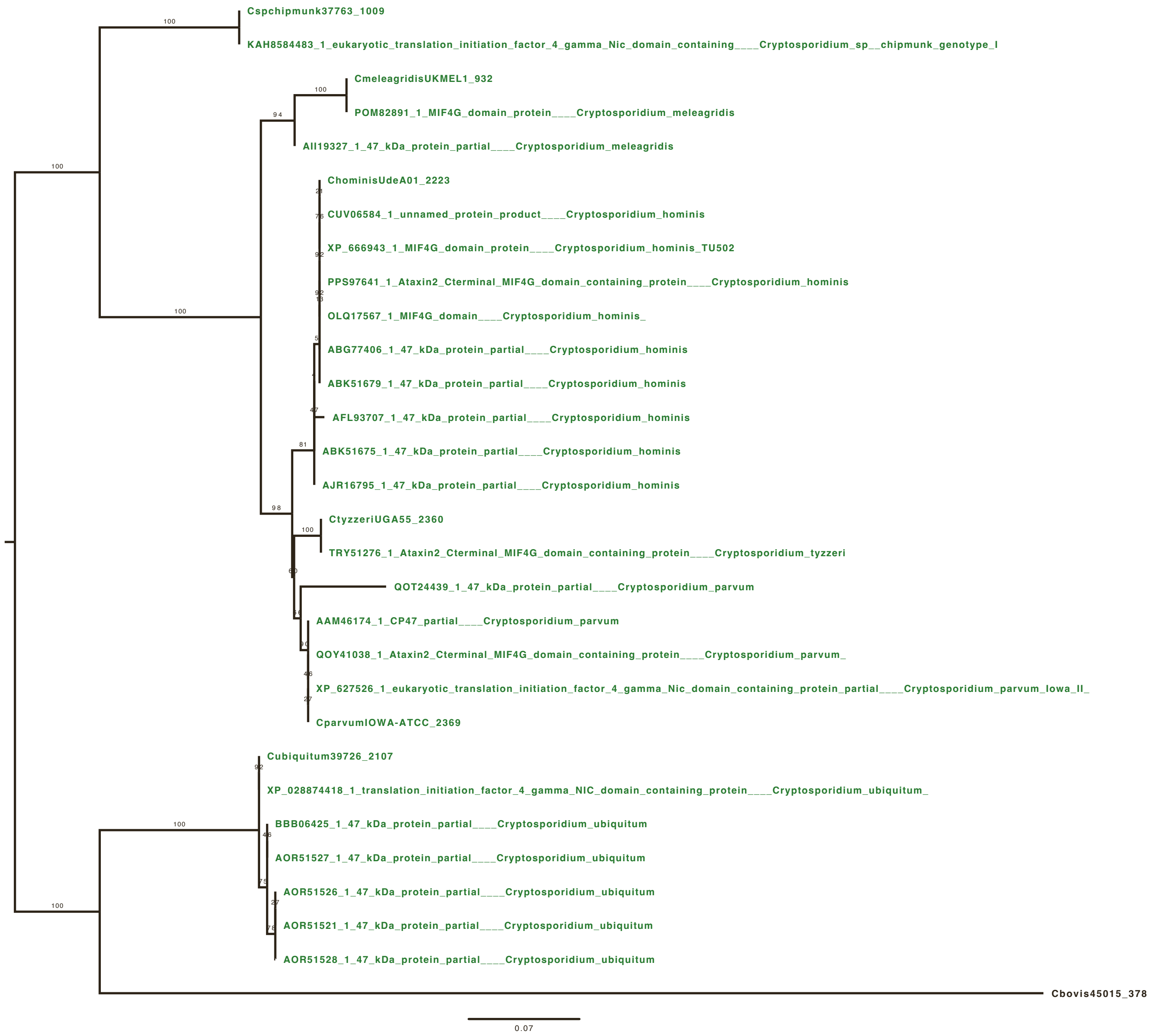

Fig. S9: Maximum likelihood phylogenetic tree of cp47 sequences.
The tree was inferred using IQ-TREE under the LG+C20+F+G model. Branch support values were estimated from 100 rapid bootstrap replicates calculated in RAxML using the PROTGAMMALG4X model. Gastric *Cryptosporidium* species are highlighted in purple, and intestinal species in green.

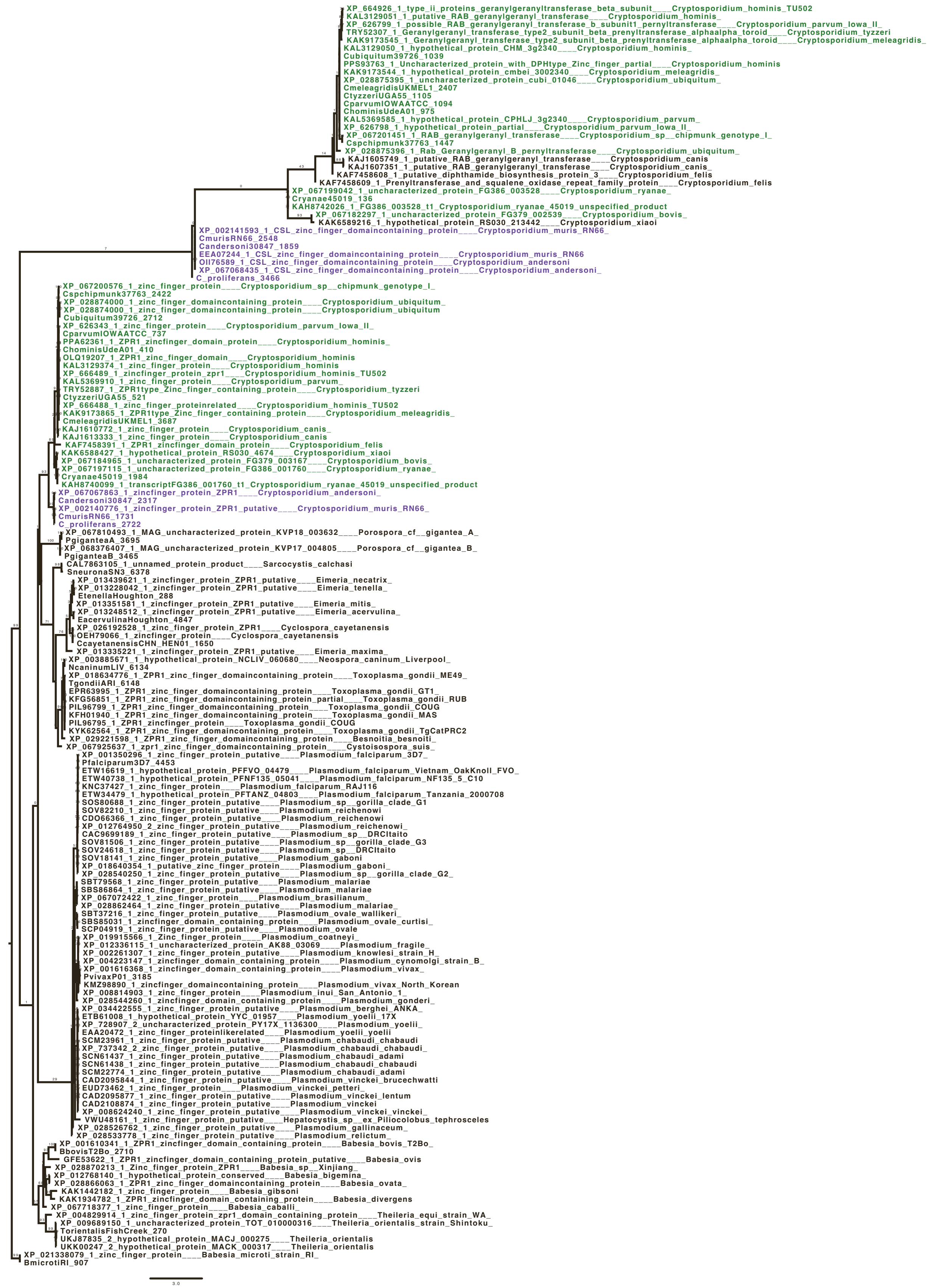

Fig. S10: Maximum likelihood phylogenetic tree of CSL zinc finger domain containing protein sequences. The tree was inferred using IQ-TREE under the LG+C20+F+G model. Branch support values were estimated from 100 rapid bootstrap replicates calculated in RAxML using the PROTGAMMALG4X model. Gastric *Cryptosporidium* species are highlighted in purple, and intestinal species in green.

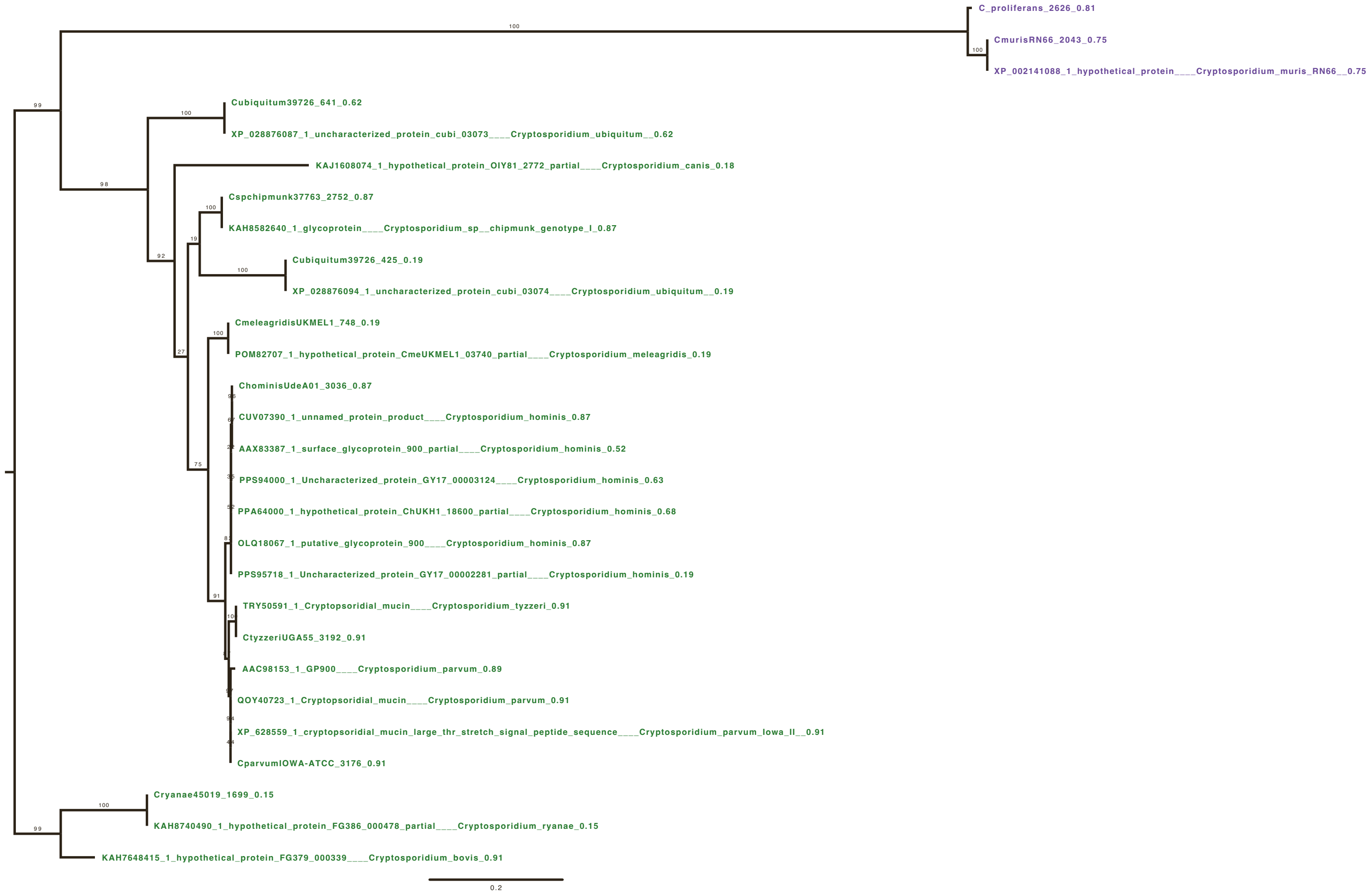

Fig. S11: Maximum likelihood phylogenetic tree of Gp900 sequences.
The tree was inferred using IQ-TREE under the LG+C20+F+G model. Branch support values were estimated from 100 rapid bootstrap replicates calculated in RAxML using the PROTGAMMALG4X model. Gastric *Cryptosporidium* species are highlighted in purple, and intestinal species in green.

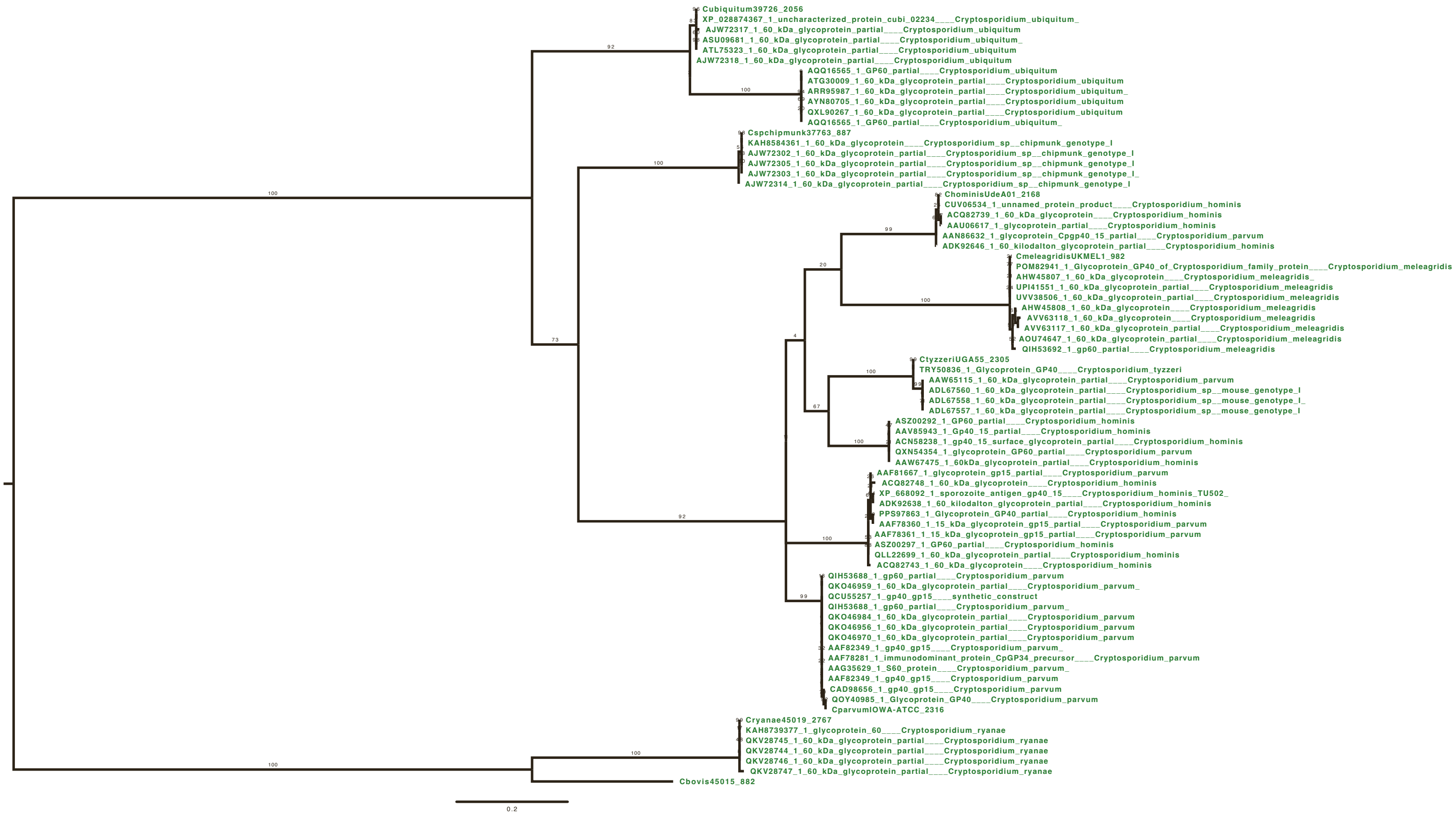

Fig. S12: Maximum likelihood phylogenetic tree of gp40/15/60 sequences.
The tree was inferred using IQ-TREE under the LG+C20+F+G model. Branch support values were estimated from 100 rapid bootstrap replicates calculated in RAxML using the PROTGAMMALG4X model. Gastric *Cryptosporidium* species are highlighted in purple, and intestinal species in green.

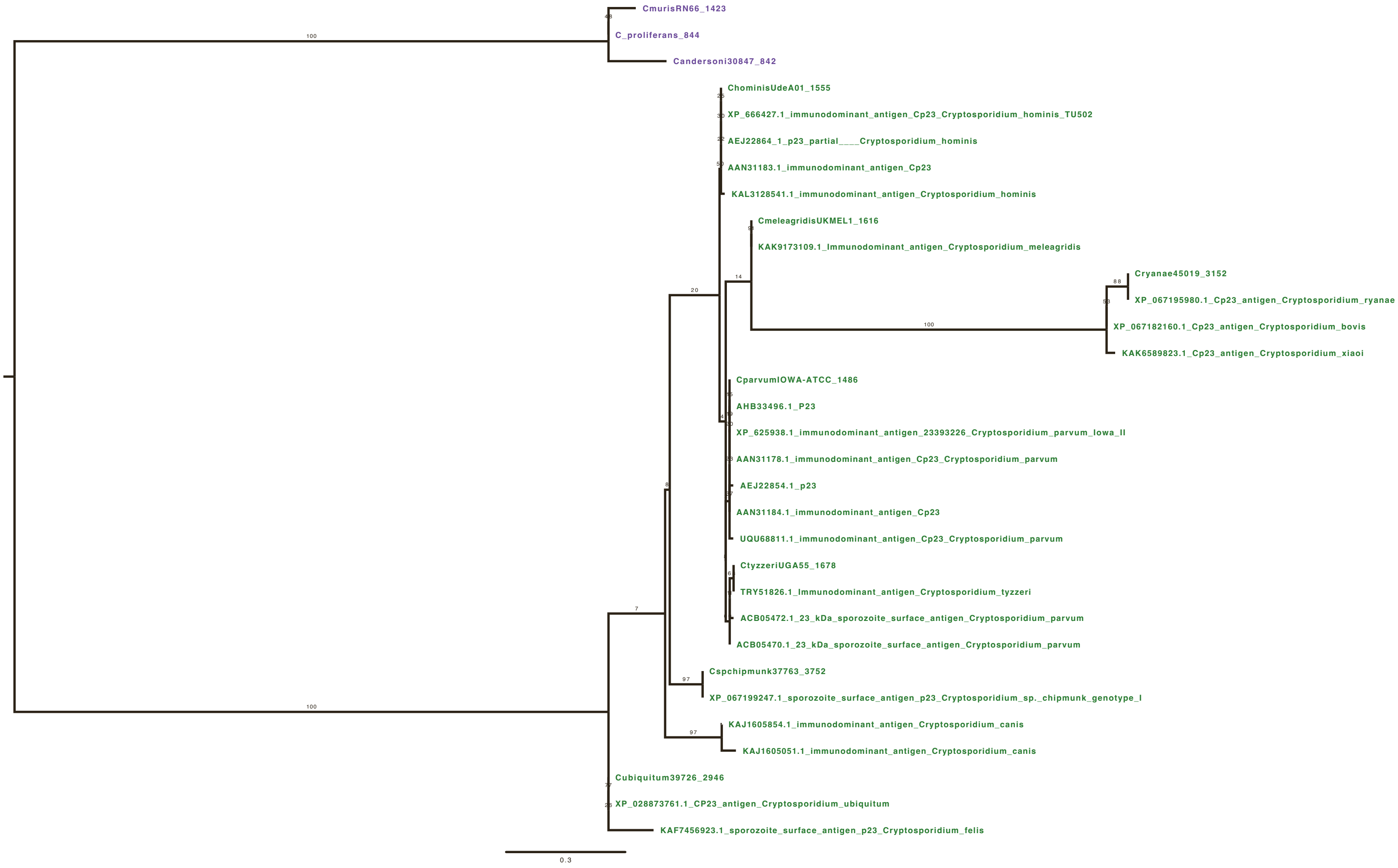

Fig. S13: Maximum likelihood phylogenetic tree of p23 sequences.
The tree was inferred using IQ-TREE under the LG+C20+F+G model. Branch support values were estimated from 100 rapid bootstrap replicates calculated in RAxML using the PROTGAMMALG4X model. Gastric *Cryptosporidium* species are highlighted in purple, and intestinal species in green.

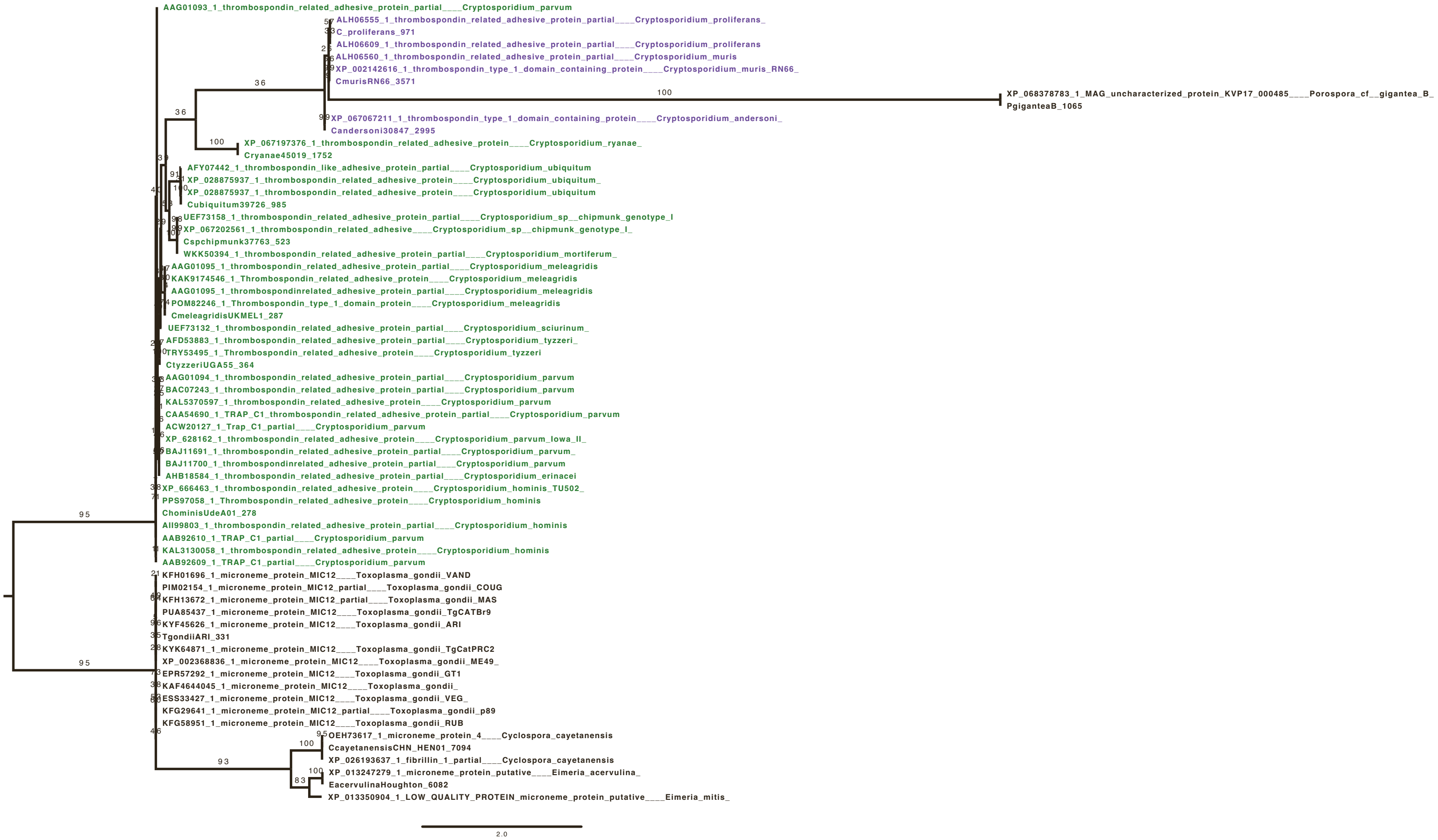

Fig. S14: Maximum likelihood phylogenetic tree of Thrombospondin-related adhesive protein C1 (TRAP-C1) sequences.

The tree was inferred using IQ-TREE under the LG+C20+F+G model. Branch support values were estimated from 100 rapid bootstrap replicates calculated in RAxML using the PROTGAMMALG4X model. Gastric *Cryptosporidium* species are highlighted in purple, and intestinal species in green.

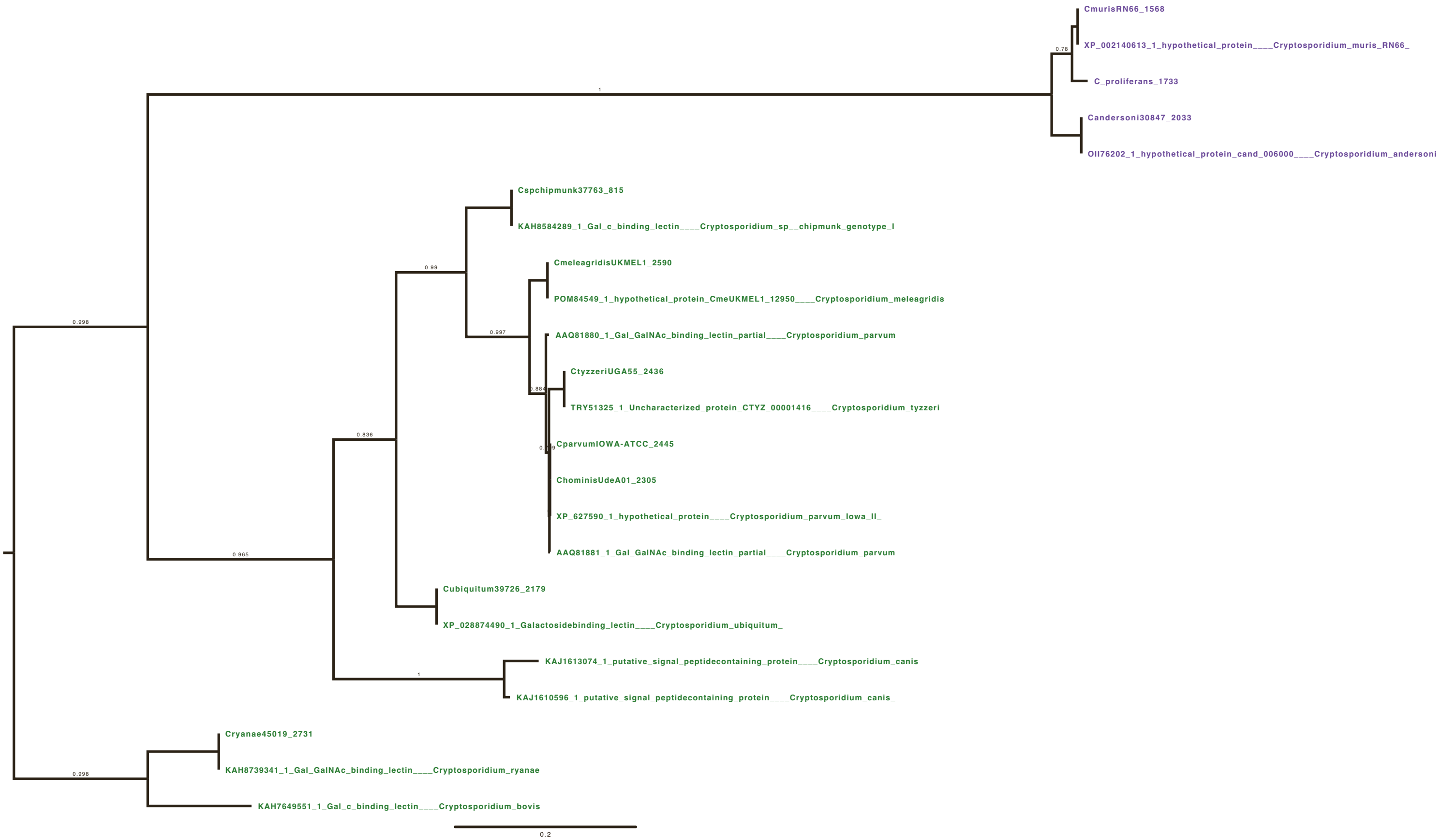

Fig. S15: Maximum likelihood phylogenetic tree of P30 sequences.
The tree was inferred using IQ-TREE under the LG+C20+F+G model. Branch support values were estimated from 100 rapid bootstrap replicates calculated in RAxML using the PROTGAMMALG4X model. Gastric *Cryptosporidium* species are highlighted in purple, and intestinal species in green.

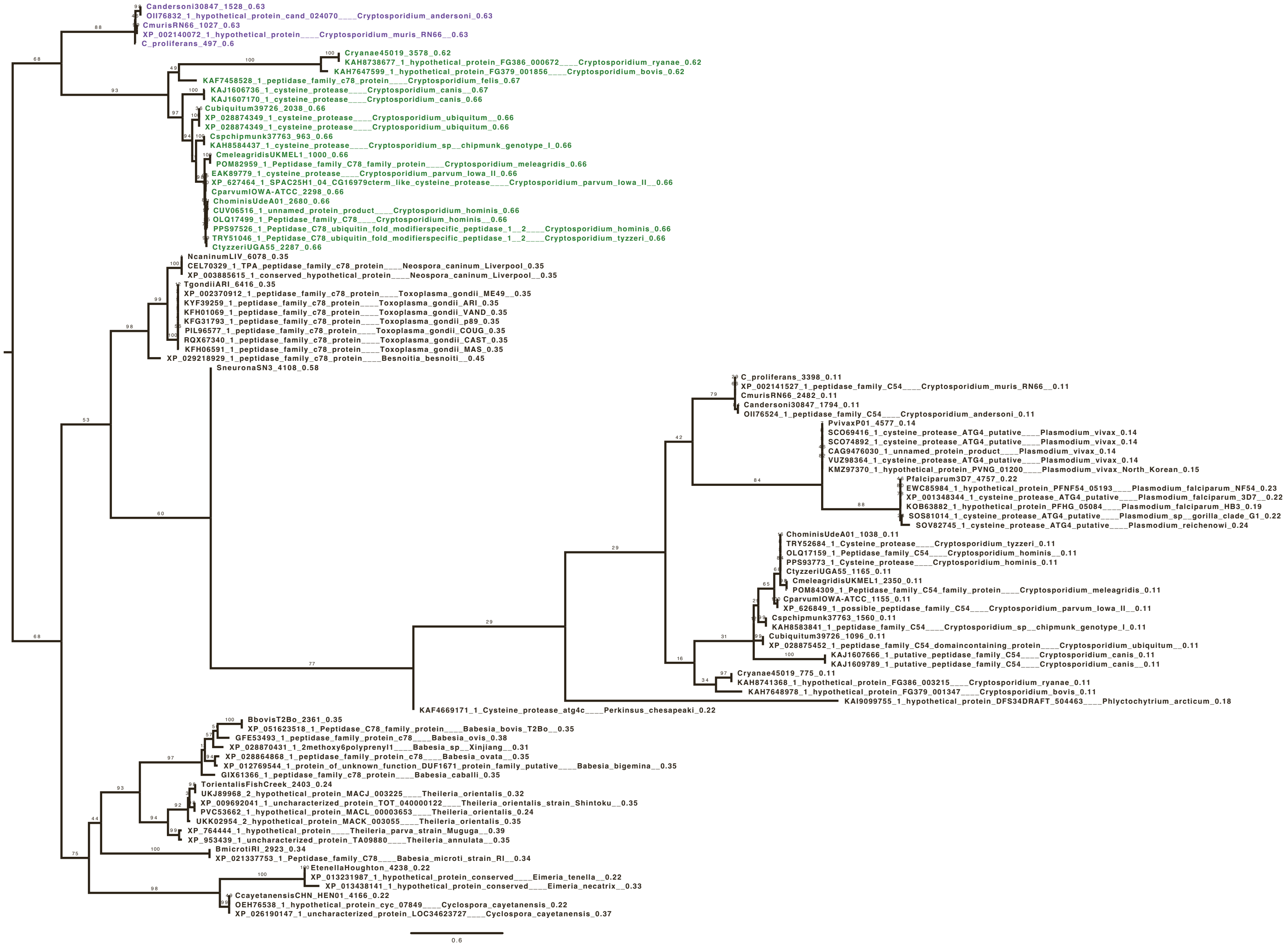

Fig. S16: Maximum likelihood phylogenetic tree of Cysteine proteases sequences.
The tree was inferred using IQ-TREE under the LG+C20+F+G model. Branch support values were estimated from 100 rapid bootstrap replicates calculated in RAxML using the PROTGAMMALG4X model. Gastric *Cryptosporidium* species are highlighted in purple, and intestinal species in green.

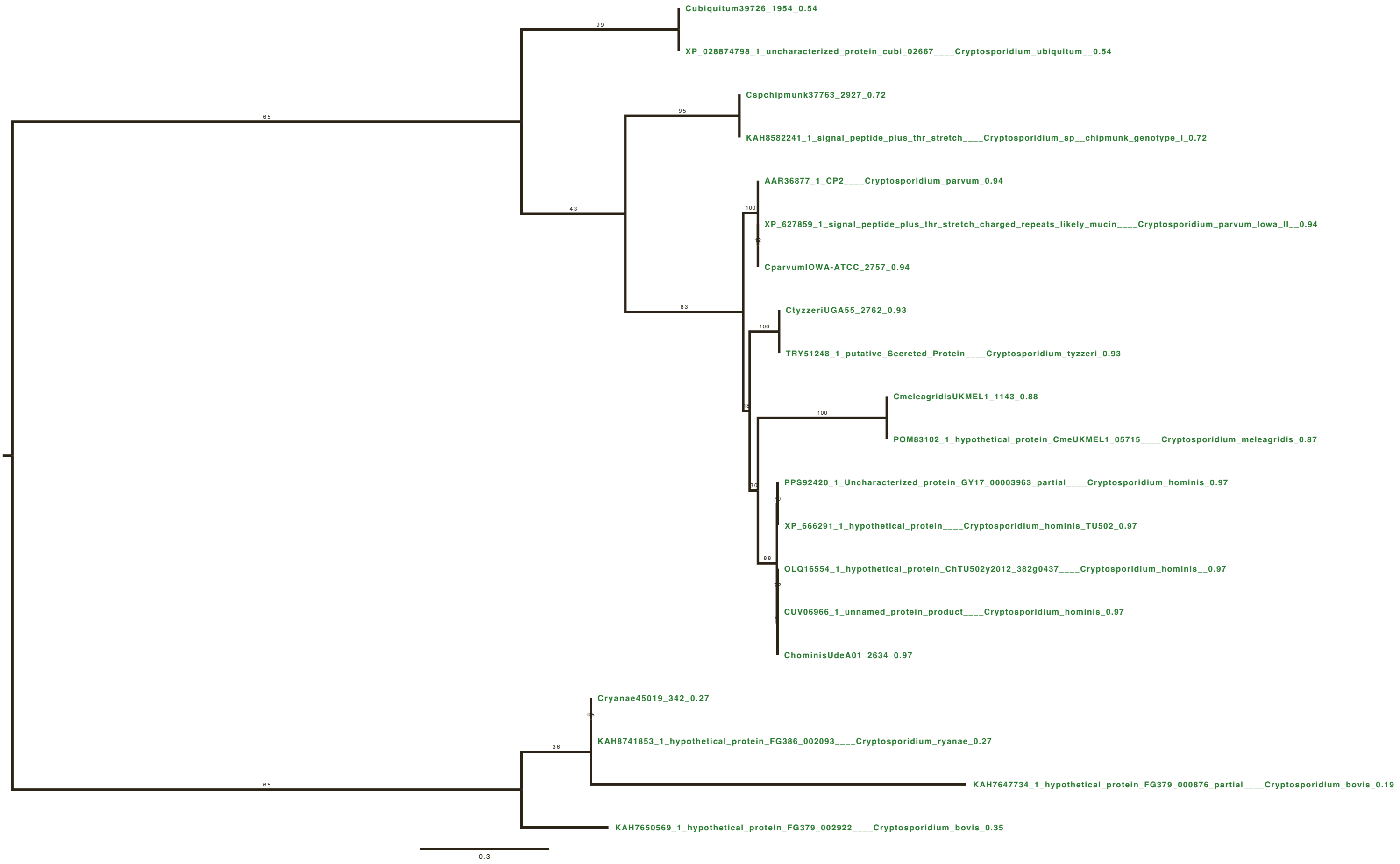

Fig. S17: Maximum likelihood phylogenetic tree of cp2 sequences.

The tree was inferred using IQ-TREE under the LG+C20+F+G model. Branch support values were estimated from 100 rapid bootstrap replicates calculated in RAxML using the PROTGAMMALG4X model. Gastric *Cryptosporidium* species are highlighted in purple, and intestinal species in green.

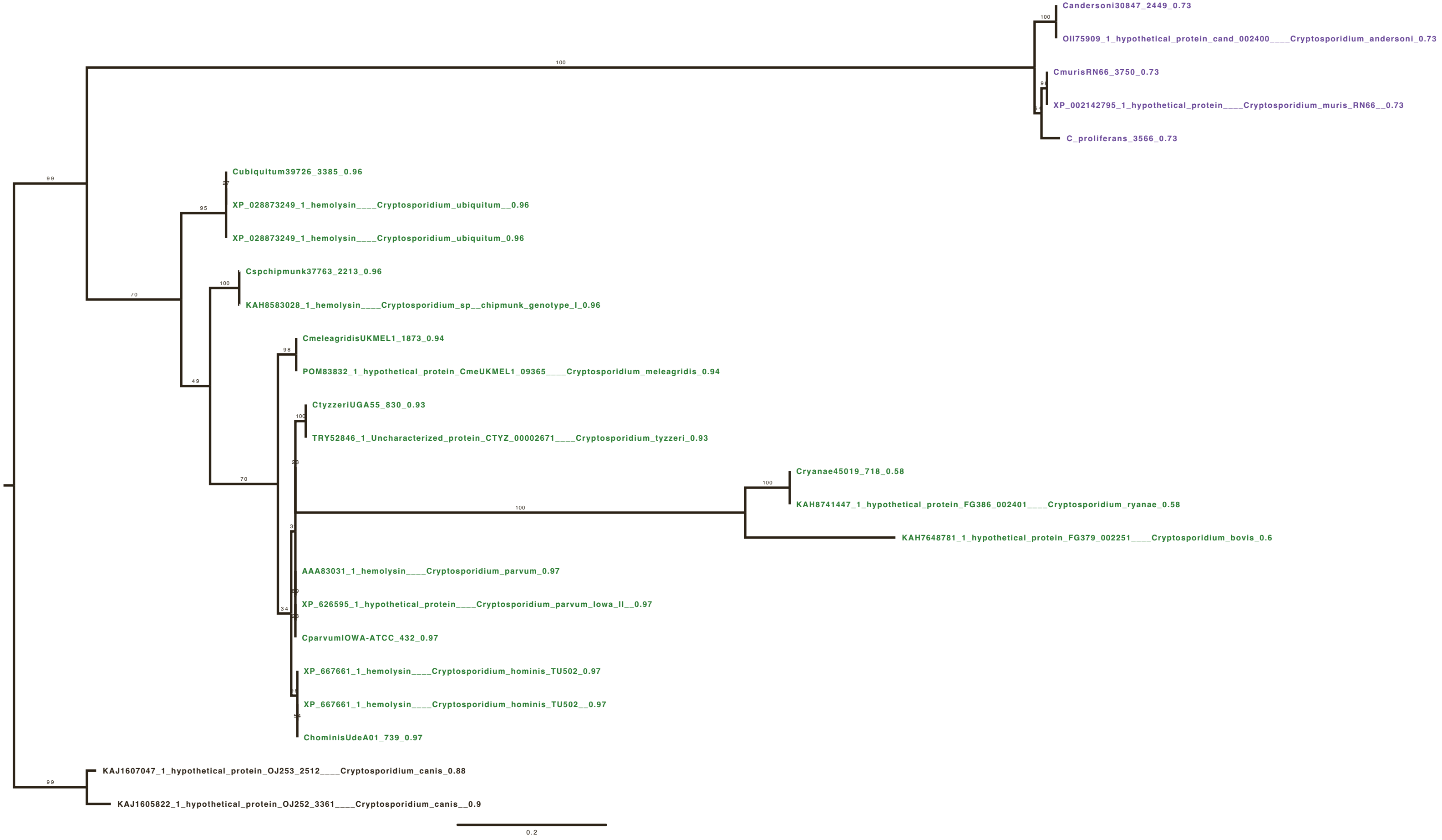

Fig. S18: Maximum likelihood phylogenetic tree of Hemolysine H4 sequences.
The tree was inferred using IQ-TREE under the LG+C20+F+G model. Branch support values were estimated from 100 rapid bootstrap replicates calculated in RAxML using the PROTGAMMALG4X model. Gastric *Cryptosporidium* species are highlighted in purple, and intestinal species in green.

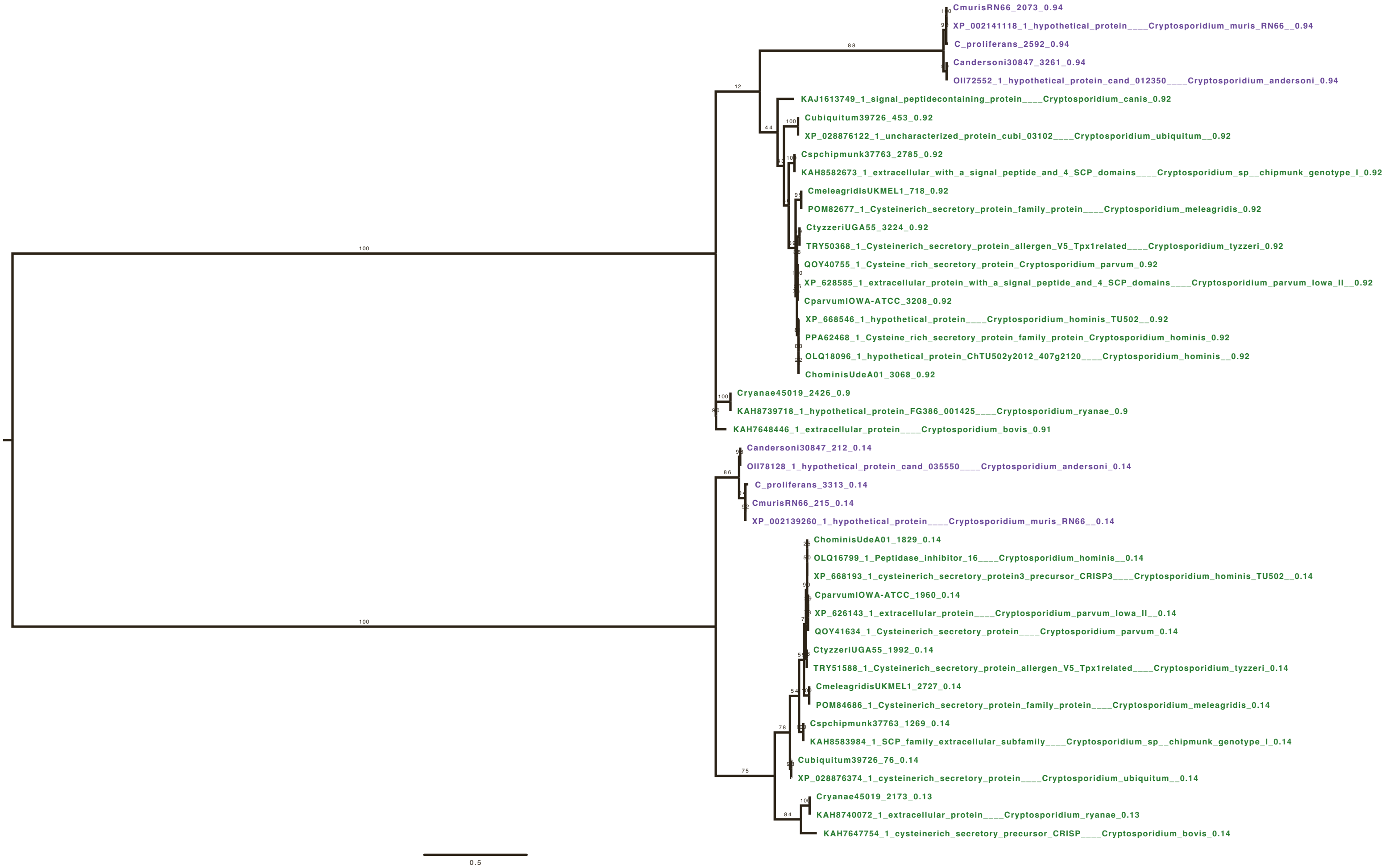

Fig. S19: Maximum likelihood phylogenetic tree of Cysteine-rich secretory protein sequences. The tree was inferred using IQ-TREE under the LG+C20+F+G model. Branch support values were estimated from 100 rapid bootstrap replicates calculated in RAxML using the PROTGAMMALG4X model. Gastric *Cryptosporidium* species are highlighted in purple, and intestinal species in green.

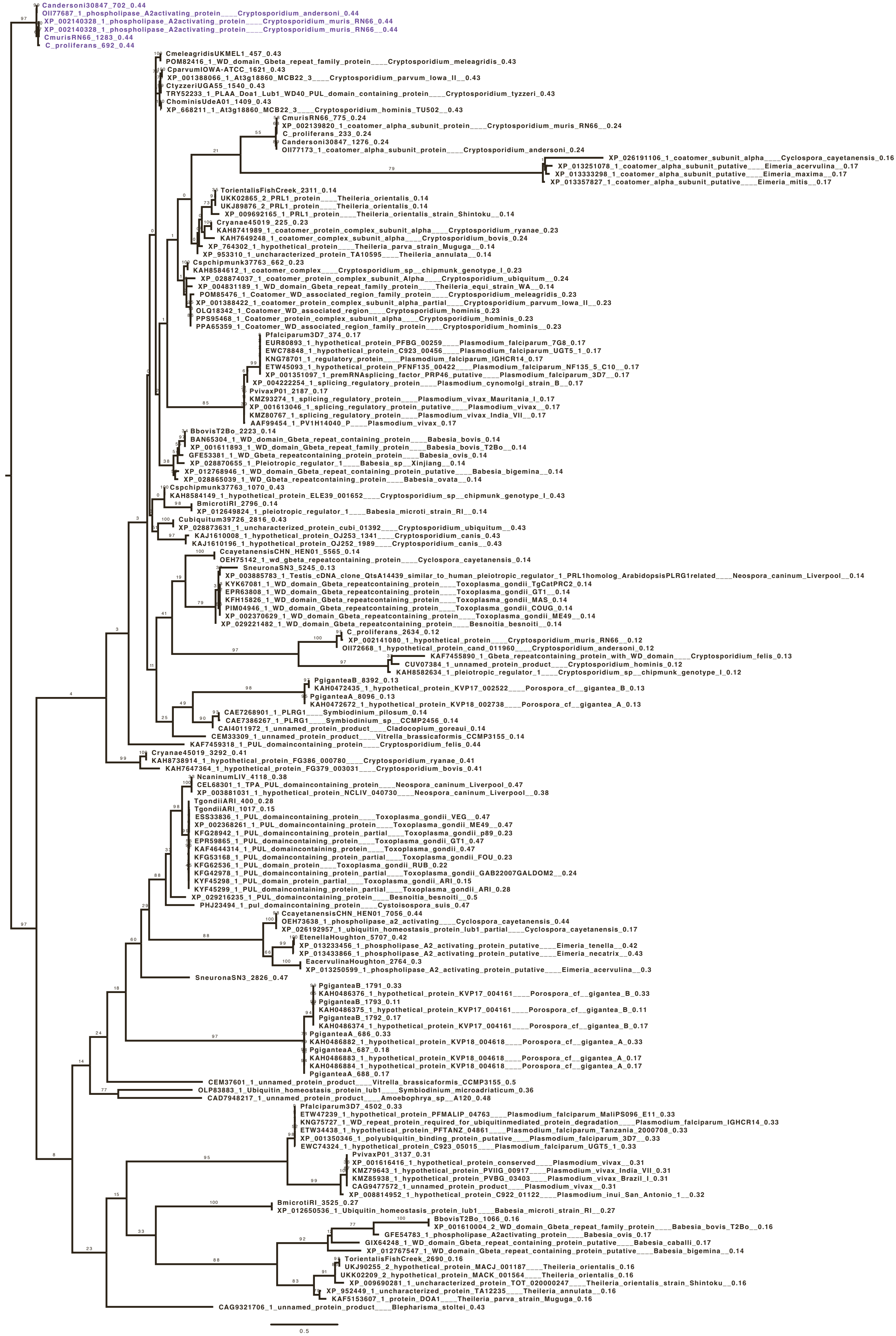

Fig. S20: Maximum likelihood phylogenetic tree of Secretory phospholipase sequences.
The tree was inferred using IQ-TREE under the LG+C20+F+G model. Branch support values were estimated from 100 rapid bootstrap replicates calculated in RAxML using the PROTGAMMALG4X model. Gastric *Cryptosporidium* species are highlighted in purple, and intestinal species in green.

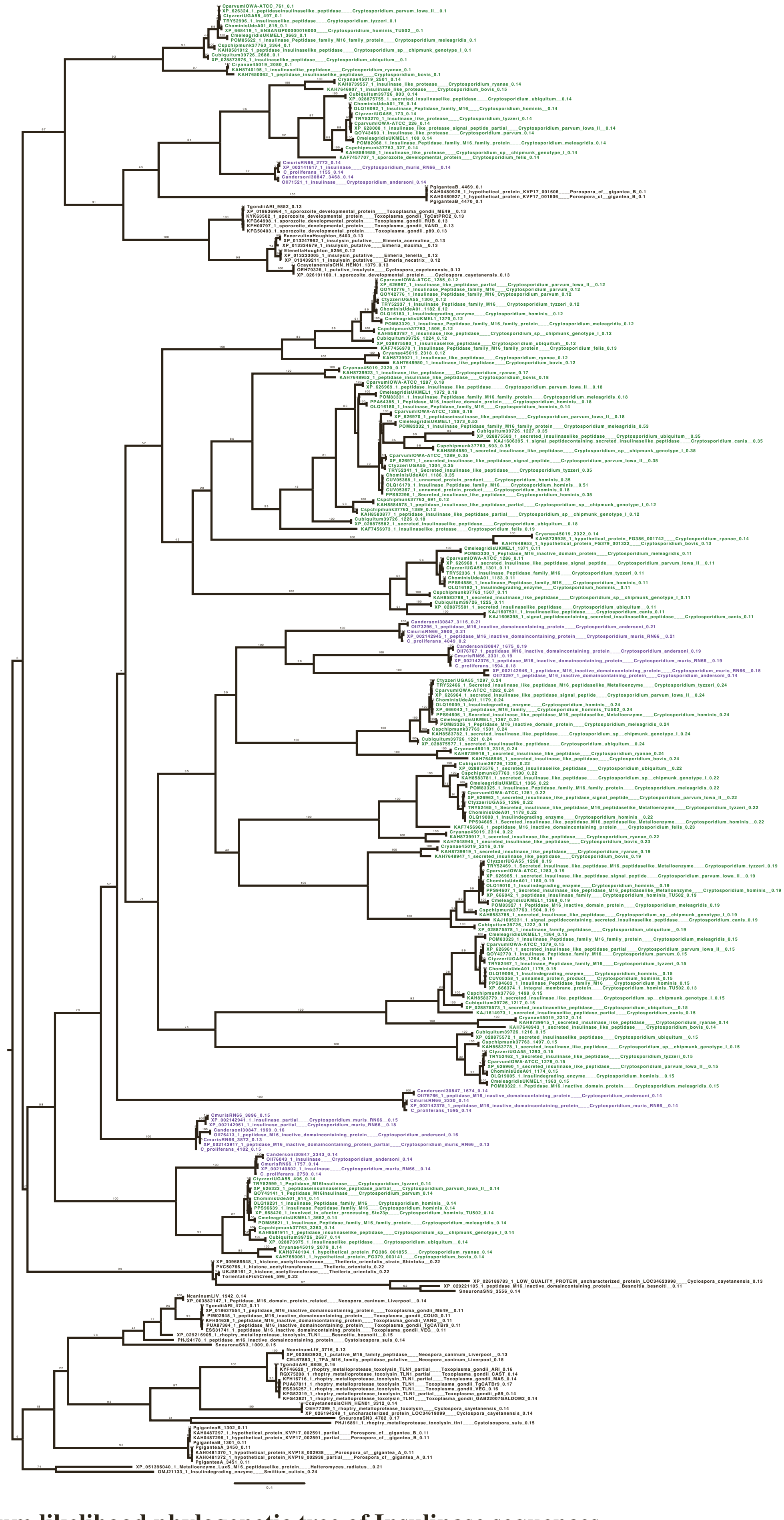

Fig. S21: Maximum likelihood phylogenetic tree of Insulinase sequences.
The tree was inferred using IQ-TREE under the LG+C20+F+G model. Branch support values were estimated from 100 rapid bootstrap replicates calculated in RAxML using the PROTGAMMALG4X model. Gastric *Cryptosporidium* species are highlighted in purple, and intestinal species in green.

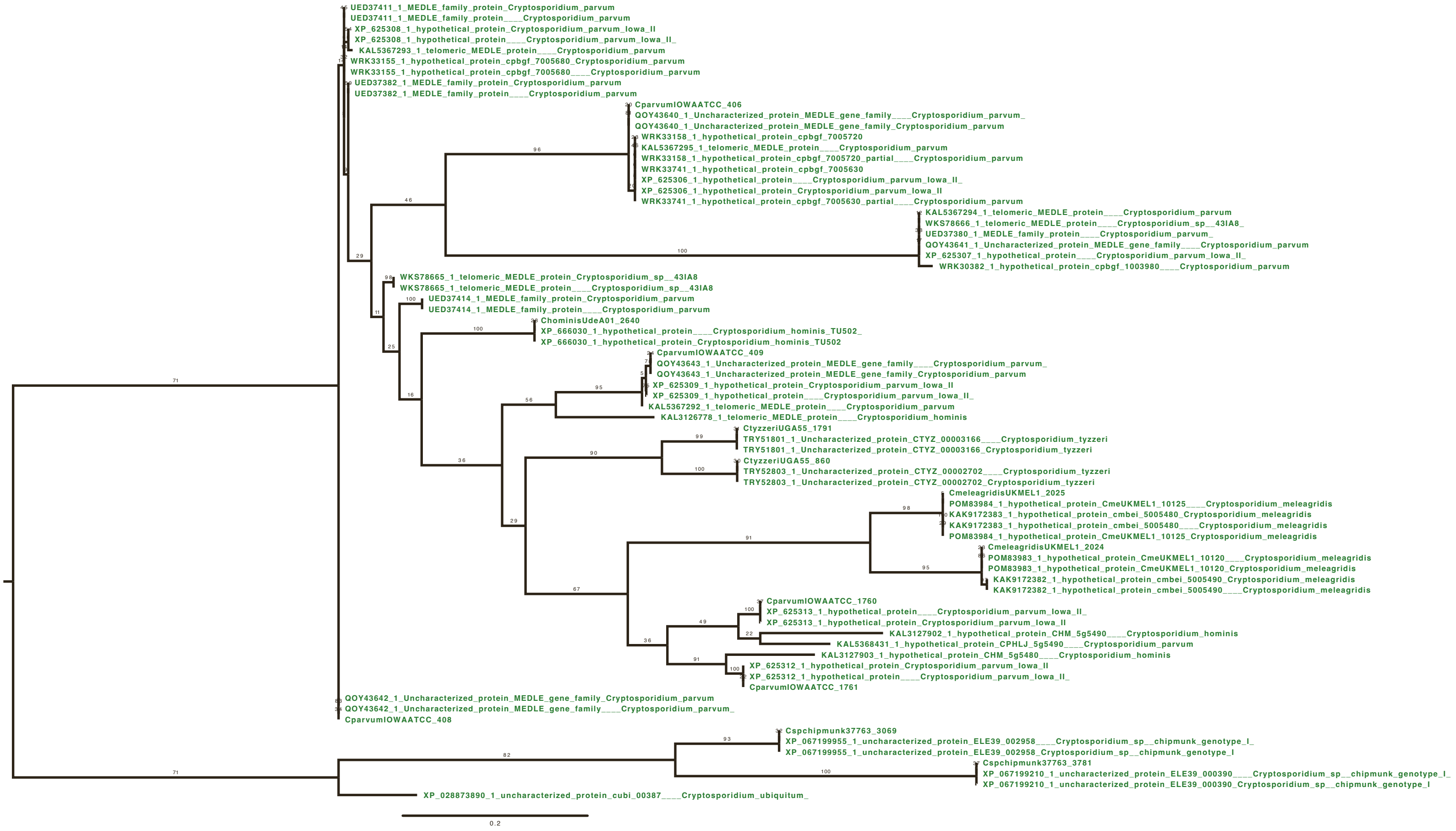

Fig. S22: Maximum likelihood phylogenetic tree of MEDLE sequences.
The tree was inferred using IQ-TREE under the LG+C20+F+G model. Branch support values were estimated from 100 rapid bootstrap replicates calculated in RAxML using the PROTGAMMALG4X model. Gastric *Cryptosporidium* species are highlighted in purple, and intestinal species in green.

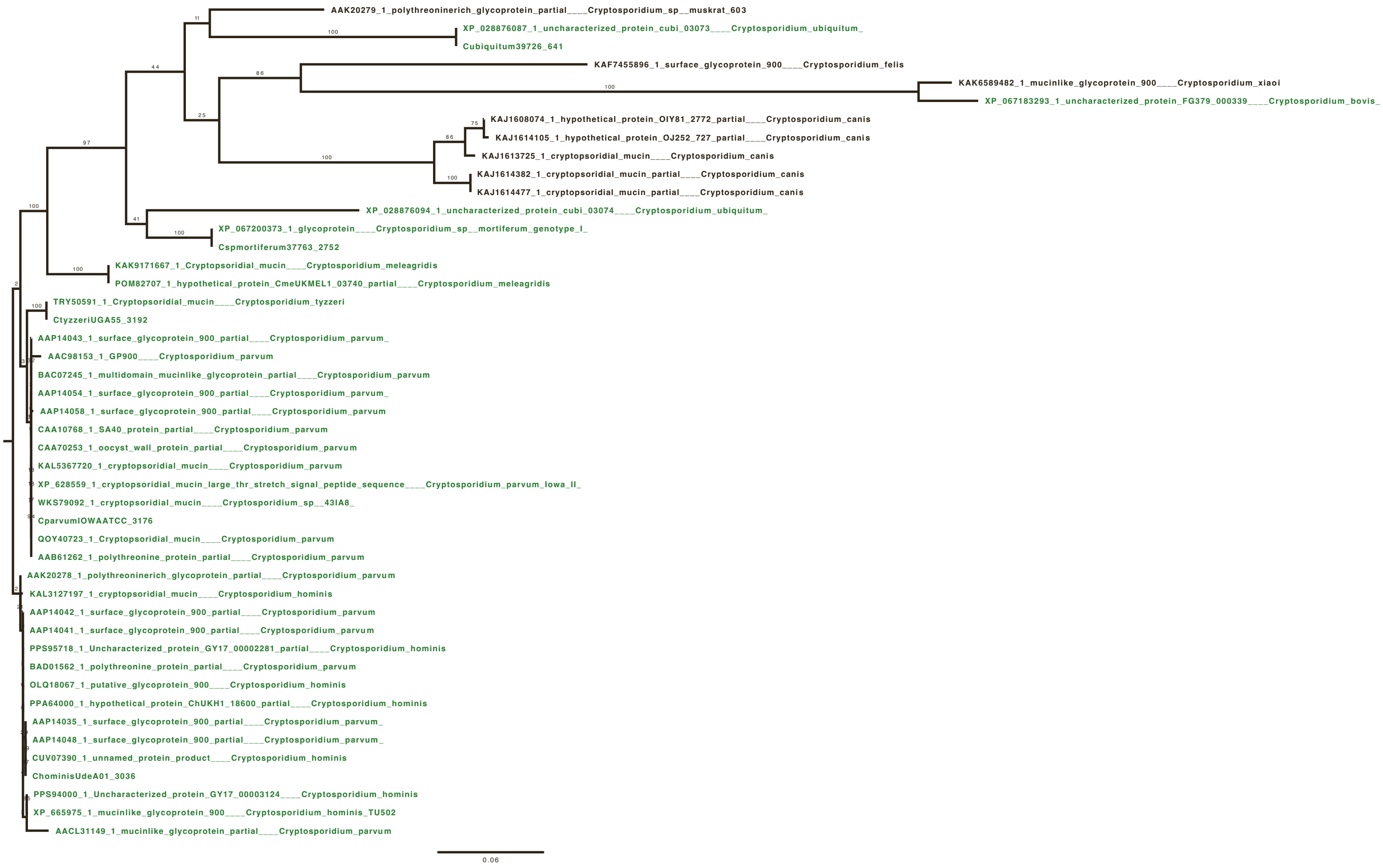

Fig. S23: Maximum likelihood phylogenetic tree of Mucin-like glycoprotein sequences.
The tree was inferred using IQ-TREE under the LG+C20+F+G model. Branch support values were estimated from 100 rapid bootstrap replicates calculated in RAxML using the PROTGAMMALG4X model. Gastric *Cryptosporidium* species are highlighted in purple, and intestinal species in green.

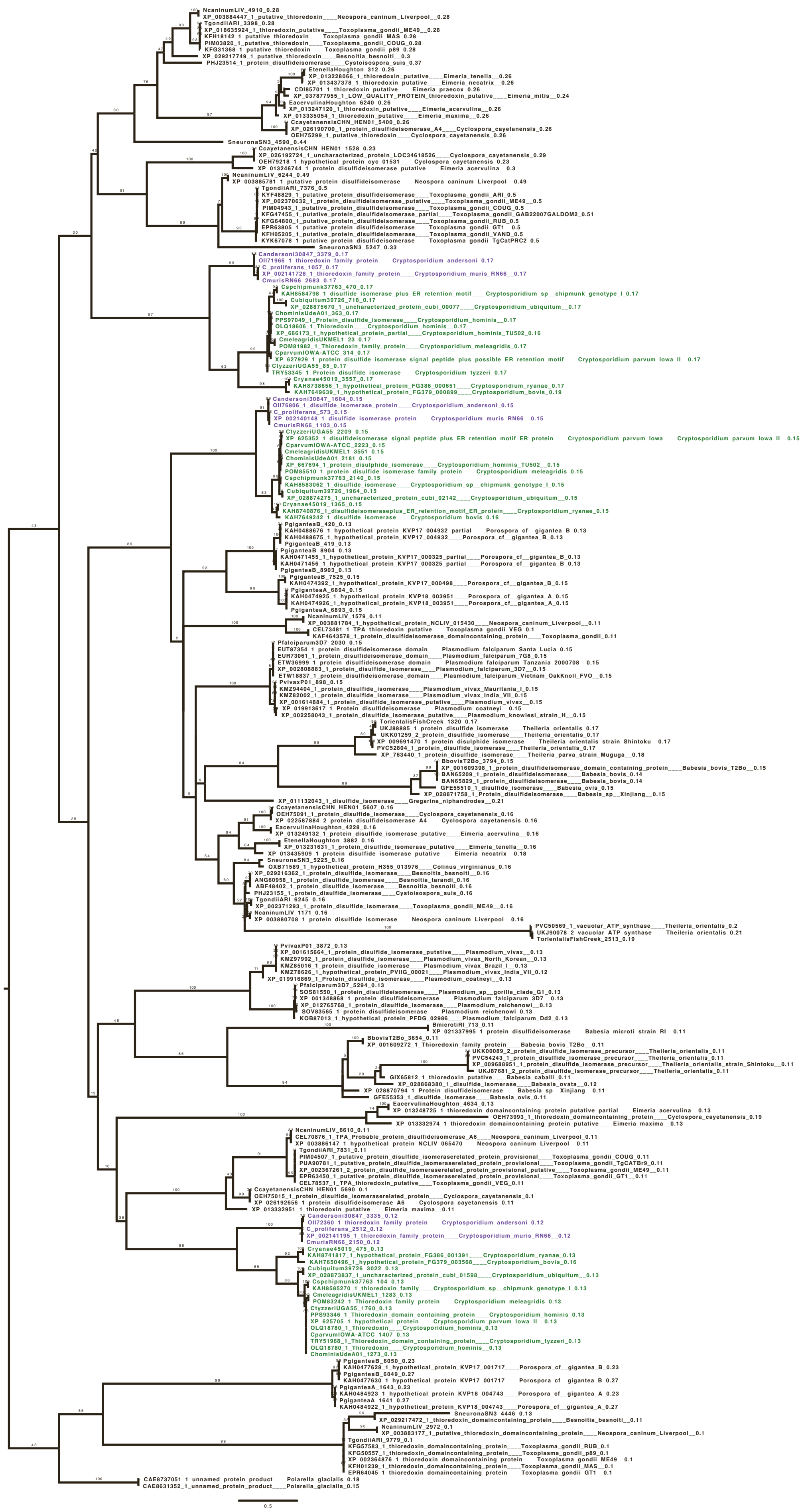

Fig. S24: Maximum likelihood phylogenetic tree of Thioredoxin sequences.
The tree was inferred using IQ-TREE under the LG+C20+F+G model. Branch support values were estimated from 100 rapid bootstrap replicates calculated in RAxML using the PROTGAMMALG4X model. Gastric *Cryptosporidium* species are highlighted in purple, and intestinal species in green.

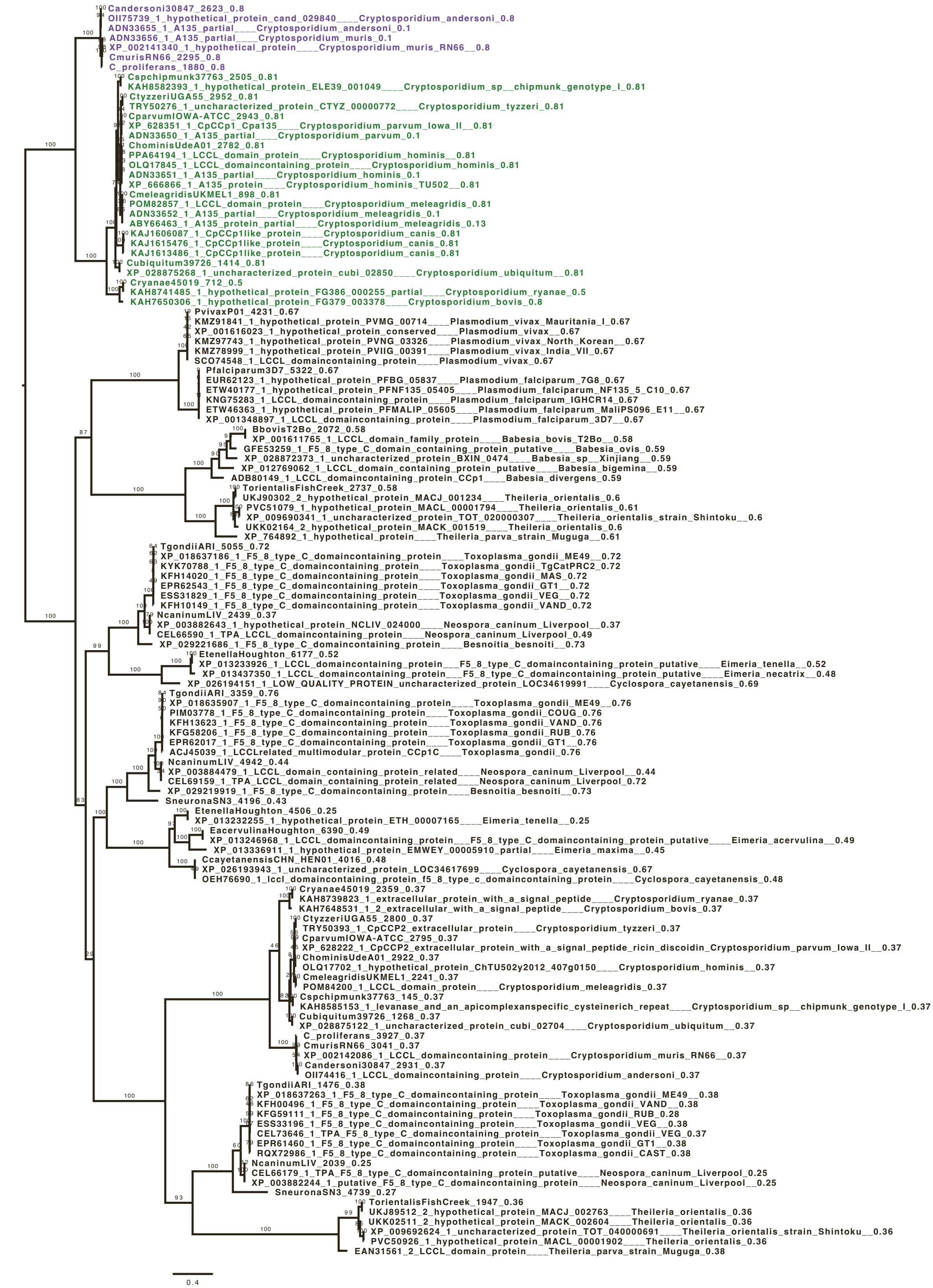

Fig. S25: Maximum likelihood phylogenetic tree of A153 sequences.
The tree was inferred using IQ-TREE under the LG+C20+F+G model. Branch support values were estimated from 100 rapid bootstrap replicates calculated in RAxML using the PROTGAMMALG4X model. Gastric *Cryptosporidium* species are highlighted in purple, and intestinal species in green.
